# Supplementary material for: Efficacy, safety and single-cell analysis of neoadjuvant immunochemotherapy in locally advanced oral squamous cell carcinoma: a phase II trial
Source: Nat Commun. 2025 Apr 28;16:3968. doi: 10.1038/s41467-025-59004-w (PMC12037888; doi:10.1038/s41467-025-59004-w)
Supplement: Supplementary file 1 — Supplementary Information [file 41467_2025_59004_MOESM1_ESM.pdf]

Efficacy, safety and single-cell analysis of neoadjuvant immunochemotherapy in locally advanced oral squamous cell carcinoma: a phase II trial

Zhongzheng Xiang[1*], Xiaoyuan Wei[1*], Zhuoyuan Zhang[2*], Yueyang Tang[3*], Linyan Chen[4*], Chenfeng Tan[1], Yuanyuan Zeng[1], Jun Wang[1], Guile Zhao[2], Zelei Dai[1], Mingmin He[1], Ningyue Xu[1], Chunjie Li[2#], Yi Li[2#] & Lei Liu[1#]

[1] Department of Head and Neck Oncology, Cancer Center & State Key Laboratory of Biotherapy, West China Hospital, Sichuan University, Chengdu, China.
[2] Department of Head and Neck Oncology & State Key Laboratory of Oral Diseases & National Clinical Research Center for Oral Diseases, West China Hospital of Stomatology, Sichuan University, Chengdu, China.
[3] Department of Oral Pathology & State Key Laboratory of Oral Diseases & National Clinical Research Center for Oral Diseases, West China Hospital of Stomatology, Sichuan University, Chengdu, China.
[4] Department of Biotherapy, Cancer Center & State Key Laboratory of Biotherapy, West China Hospital, Sichuan University, Chengdu, China.

[*]These authors contributed equally.

[#] Correspondence to:
Lei Liu, Department of Head and Neck Oncology, Cancer Center & State Key Laboratory of Biotherapy, West China Hospital, Sichuan University, Chengdu, China.
Email: liuleihx@gmail.com
Yi Li, Department of Head and Neck Oncology & State Key Laboratory of Oral Diseases & National Clinical Research Center for Oral Diseases, West China Hospital of Stomatology, Sichuan University, Chengdu, China.
Email: liyi1012@163.com
Chunjie Li, Department of Head and Neck Oncology & State Key Laboratory of Oral Diseases & National Clinical Research Center for Oral Diseases, West China Hospital of Stomatology, Sichuan University, Chengdu, China.
Email: lichunjie@scu.edu.cn

Doses modifications for neoadjuvant immunochemotherapy

Camrelizumab was suspended for grade 3 reactive capillary hyperplasia, thrombocytopenia, neutropenia, anemia, grade 2 pneumonia, hepatitis, myocarditis, adrenal insufficiency, grade 2-3 diarrhea, nephritis, hypothyroidism, hyperthyroidism, pancreatitis, etc. Camrelizumab was resumed when these adverse events resolved to grade ≤1. Permanent discontinuation of camrelizumab was required for grade 3-4 pneumonia, hepatitis, adrenal insufficiency, myocarditis, grade 4 hypothyroidism, hyperthyroidism, reactive capillary hyperplasia, diarrhea, nephritis, etc. For patients experiencing grade 3-4 diarrhea, hepatitis, nephritis, thrombocytopenia, neutropenia, anemia, etc, the doses of albumin paclitaxel and cisplatin were reduced by 25%-50%.

Radiotherapy protocol

Radiotherapy was performed within 6 weeks after surgery. The recommended radiation dose was 54-60 Gy (1.8-2.0 Gy perday, 5 days perweek, for 6 weeks). In patients with positive resectional margin and/or extranodal extension, cisplantin-based concurrent chemoradiotherapy was recommended, and a total of 66 Gy was allowed. A 20% dose reduction of cisplantin was required for patients experiencing grade 3 neutropenia, grade 2 thrombocytopenia, creatinine clearance of 40–60 mL/min, or grade 2 neurotoxicity in concurrent chemoradiotherapy phase. Radiotherapy was suspended for grade 4 oral mucositis, radiodermatitis, or radiation-induced myelosuppression.

Single-cell RNA-seq data preprocessing

Pathway analysis

Differentially expressed genes (DEGs) were selected using the function FindMarkers (test.use = presto). p value < 0.05 and $|\log_2 \text{foldchange}| > 0.58$ was set as the threshold for significantly differential expression. GO enrichment pathway enrichment analysis were analyzed by R package VISION (version 3.0.1).

Pseudotime analysis by Monocle

Single-cell trajectory analyses were carried out using the Monocle package (version 2.18.0). The starting subject CellDataSet class was constructed through a normalized expression cells × genes matrix. The DEGs among subclusters were validated using *differentialGeneTest* function and then utilized to determine cell progression. DDRTree, included in the *reduceDimension* function, was used for data dimension reduction. Then, cells were sorted in pseudotime by the *orderCells* function. Trajectories were visualized in two-dimensional space using the *plot_cell_trajectory* function.

Cell-cell communication analysis

A tailored tool for single-cell transcriptome data, CellChat (v 1.1.0), was used to perform cell-cell communication analysis on the basis of manually designed repository of ligand and receptor interactions. Briefly, the method speculated underlying cell-cell interactions according to the expression of ligand-receptor pairs that interacted between two clusters. For inclusion in downstream analyses, ligand or receptor gene expression was required in more than 30% of cells in each cluster. To assess the significance of a ligand-receptor pair between two clusters, cluster labels for each cell were randomly assigned 1,000 times for permutation test. An average expression of a specific ligand and receptor pair in two clusters among the 1,000 permutated results was used to calculate an empirical p value.

Processing of T cell receptor (TCR) scRNA-seq data

TCR reads were calibrated to the hg38 reference genes. TCR was annotated by 10x cellranger vdj pipeline. The clonotype of each T-cell was identified by the paired alpha and beta CDR3 sequences. Clonal TCRs were validated if at least two cells shared an identical productive alpha-beta pair. Clonotypes ≥ 2 cells were defined as amplified clonotypes. Then, differentially expanded clonotypes after treatment were further categorized as emergent and none-emergent clones according to their fractions in pre-NAIC and post-NAIC samples. Emergent clonotypes refered to MPR expanded clonotypes detected only in the post-NAIC tumor samples, whereas non-emergent clonotypes was presented in both pre- and post-NAIC tumor samples.

Cell analysis from multiplex immunofluorescence (mIF) image

The stained mIF slides were scanned by using Vectra Polaris Software (Akoya Biosciences). Then, QuPath-0.4.3 software was used for image reading and cell analysis, including cell type, quantity and location [1].

G-cross function was used for cell spatial analysis by identifying the probability distribution of one cell type (*a*) nearest to another cell type (*b*) in any given distance with a radius of *r* (μm) [2]. The G-cross function was calculated by the following formula (the term λ_b is the overall density of cells of type *b* in the slide):

$G_{ab}(r)= 1 - e^{-\lambda_b \pi r^2}$

Jaccard index was also calculated to assist in estimating the spatial distribution of varieties of cell types.

Animal experiments

Tumor model was established by subcutaneous injection of 100 μL single-cell suspensions of SCC7 cells (1×10^6 cells/mouse) on the right flank of mice. To explore which agent in NAIC had immunostimulatory or immunosuppressive effects, mice were intraperitoneally injected with saline (control), nab-paclitaxel (200 μg per mouse, Hengrui Pharmaceuticals Co. Ltd, China), cisplatin (40 μg per mouse, Yuanye, China, CAS: 15663-27-1), anti-mouse PD-1 (200 μg per mouse, STARTER, China, CAS: S0B0594), nab-paclitaxel+cisplatin, anti-mouse PD-1, nab-paclitaxel+cisplatin+anti-mouse PD-1 in 7 and 10 days after tumor cell injection. To validate the function of CXCL13, mice were intraperitoneally injected with saline (control), rmCXCL13, nab-paclitaxel+cisplatin+anti-mouse PD-1, nab-paclitaxel+cisplatin+anti-mouse PD-1+rmCXCL13 (rmCXCL13, 5 μg per mouse, R&D, catalog No. 470-BC-025/CF). Tumor volume was calculated every 3 days by the following formula: (length×width^2/2) (mm^3). Mice were euthanized through cervical dislocation 21 days after tumor implantation, and tumors were harvested for weight measurement, H&E staining, flow cytometry and multiplex immunofluorescence analyses. For flow cytometry, in a parallel experiment, on day 6

after treatment, the harvested tumors were cut up and digested into single cell suspension. After red blood cell lysis, cells were stained with anti-CD45 (1:100, Absin, abs182374-100T), anti-CD3 (1:100, BD Pharmingen, 563024), anti-CD4 (1:100, BD Pharmingen, 566407), anti-CD8 (1:100, BD Pharmingen, 563234), anti-CXCL13 (1:100, Thermo, 17-7981-82), and anti-CD19 (1:100, BD Pharmingen, 562701). Then, a flowcytometer (NovoCyte Advanteon Flow Cytometer, Agilent Technologies, Inc.) was used to analyze the stained cells. For multiplex immunofluorescence, 7-color mIF staining kit (Akoya OPAL Polaris 7-Colour Automation IHC kit) was used to evaluate the infiltration of T and B lymphocytes. Formalin-fixed, paraffinembedded (FFPE) tumor slides were deparaffinized. First antibodies, anti-CD4 (1:200, CST, 25229), anti-CD8 (1:1000, Abcam, 217344), and anti-CD20 (1:100, Invitrogen, PA5-16701), were added to the slides and incubated for one hour. Second antibodies were subsequently added and followed by Fluorophore-conjugated TSA® Plus Amplification Reagent. Meanwhile, the survival status of mice after treatment was also observed in the parallel experiments. Vectra Polaris Software (Akoya Biosciences) were used for collecting multiplex fluorescence immunohistochemistry data. QuPath-0.4.3 was used for multiplex immunofluorescence data analysis.

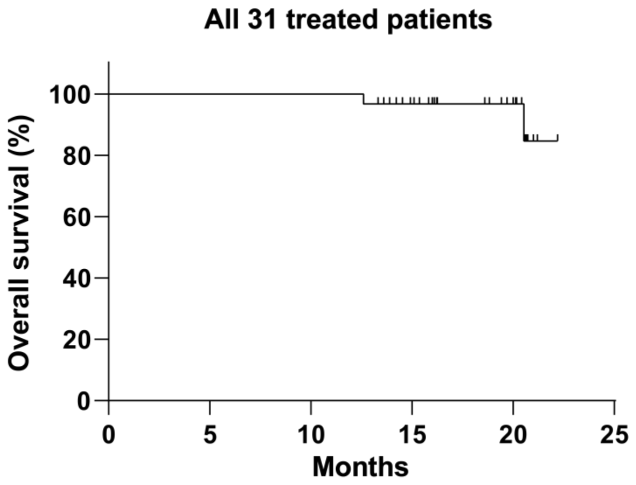

Supplementary Figure 1. Keplan-Meier analysis of overall survival for all 31 treated patients. Source data are presented as a Source Data file.

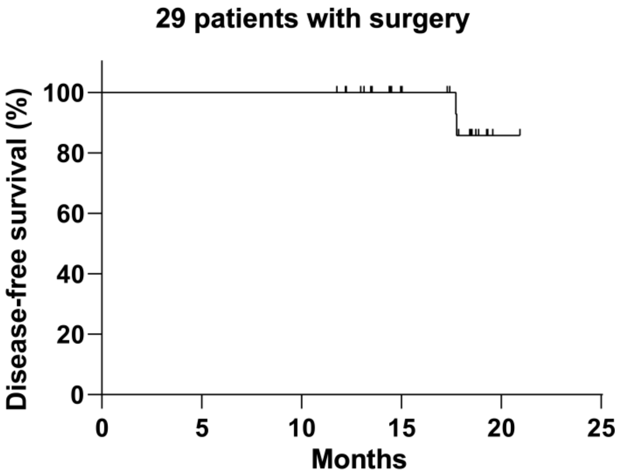

Supplementary Figure 2. Keplan-Meier analysis of disease-free survival for 29 patients who received neoadjuvant immunochemotherapy and surgery. Source data are presented as a Source Data file.

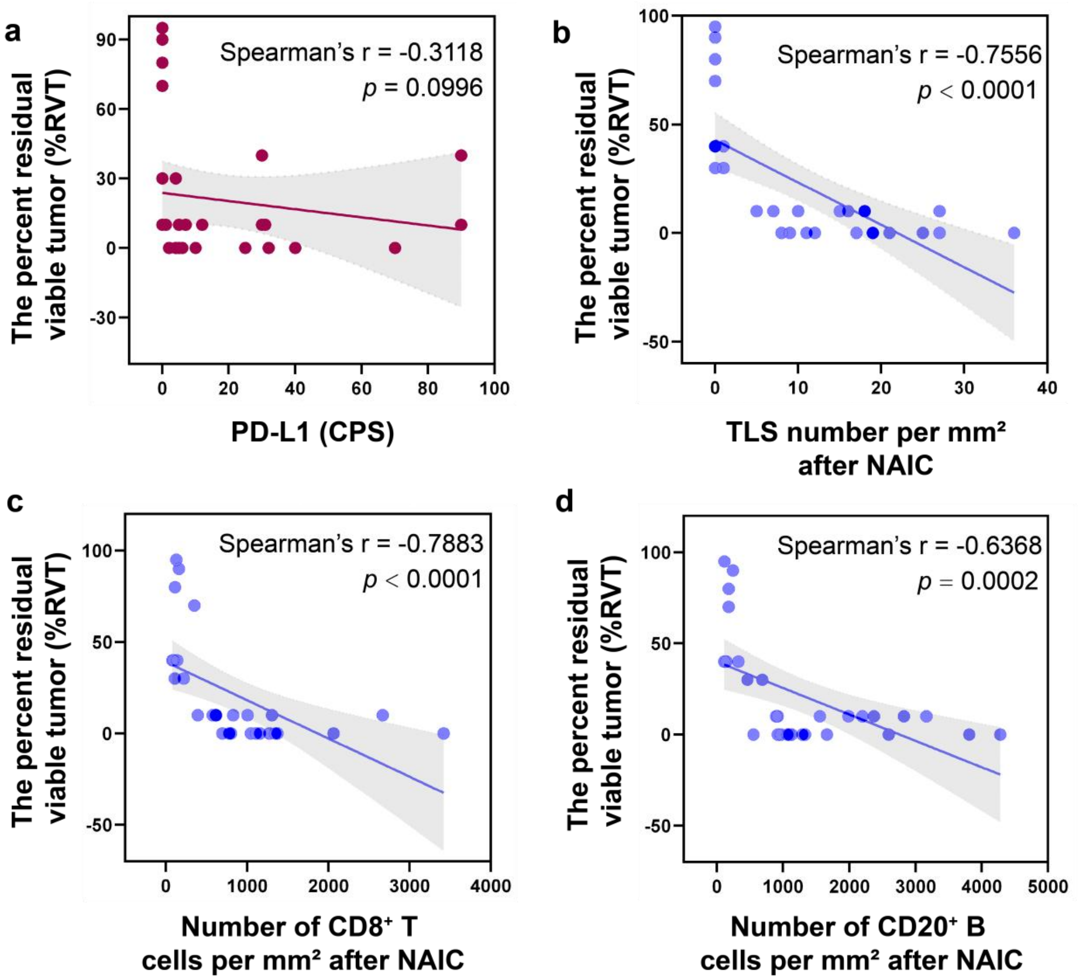

Supplementary Figure 3. Correlation between different factors and %RVT by Spearman correlation analysis. a, Correlation between PD-L1 (CPS) and %RVT (Spearman r = -0.3118, p = 0.0996). b, Correlation between TLS and %RVT after NAIC (Spearman r = -0.7556, p < 0.0001). c, Correlation between $CD8^+$ T cells density and %RVT after NAIC (Spearman r = -0.7883, p < 0.0001). d, Correlation between $CD20^+$ cells and %RVT after NAIC (Spearman r = -0.6368, p = 0.0002). Gray area represent 95% confidence interval. Two-sided test was used for statistical analysis. %RVT, percent of residual viable tumor; TLS, tertiary lymphoid structures; NAIC, neoadjuvant immunochemotherapy; CPS, combined positive score. Source data are presented as a Source Data file.

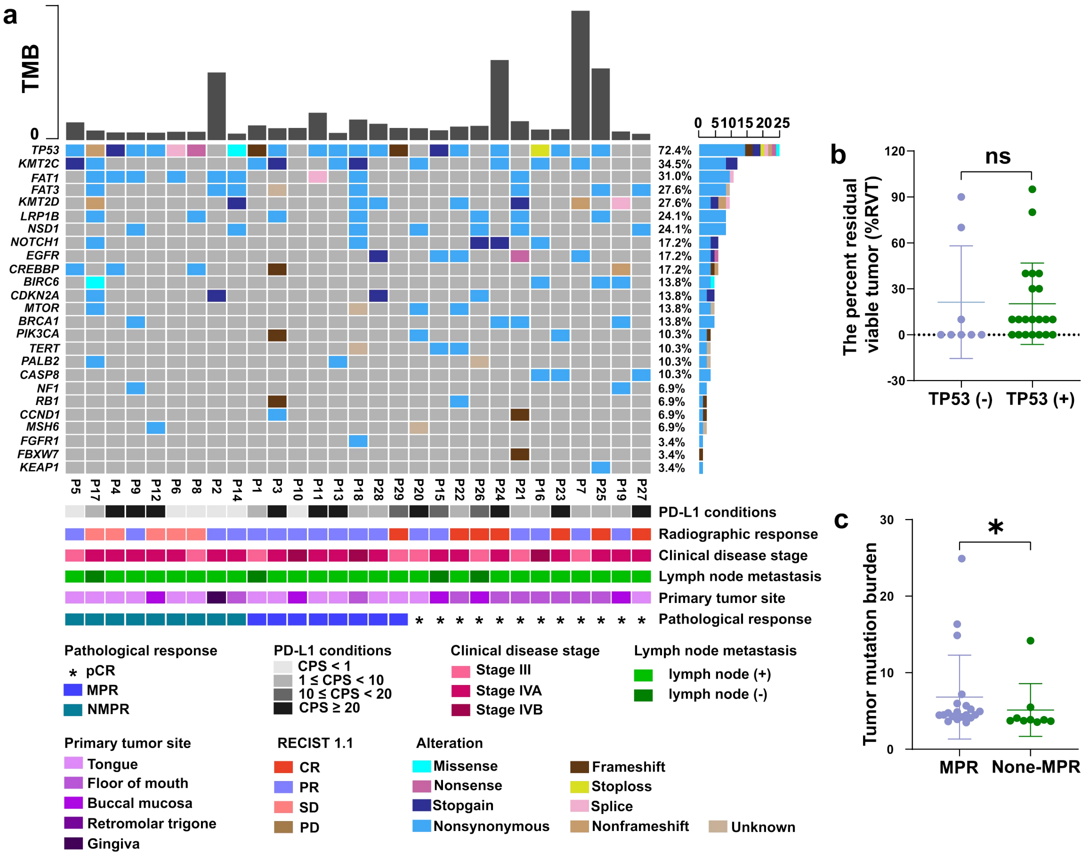

Supplementary Figure 4. Gene characteristics. a, Waterfall plot of WES in baseline primary tumor tissue. Each column represents a single patient, with gene mutation frequencies shown on the left and percentages displayed on the right. The bottom bars illustrate clinical and pathological characteristics. b, Comparison of percent of residual viable tumor (%RVT) in TP53(+) (n = 21) and TP53(-) (n = 8) populations. A two-sided Mann-Whitney U test was used for statistical analysis. Bars represent the mean with SD. The dot represents an individual data point. c, Comparison of tumor mutation burden in MPR (n = 20) and NMPR (n = 9) population. A two-sided Mann-Whitney test was used for statistical analysis. Bars represent the mean with SD. The dot represents an individual data point. TMB, tumor mutation burden; MPR, major pathologic response; NMPR, none-major pathologic response; CPS, combined positive score; CR, complete response; PR, partial response; SD, stable disease; PD, progressive disease. ns, not significant, * $P < 0.05$. Source data and p value are presented as a Source Data file.

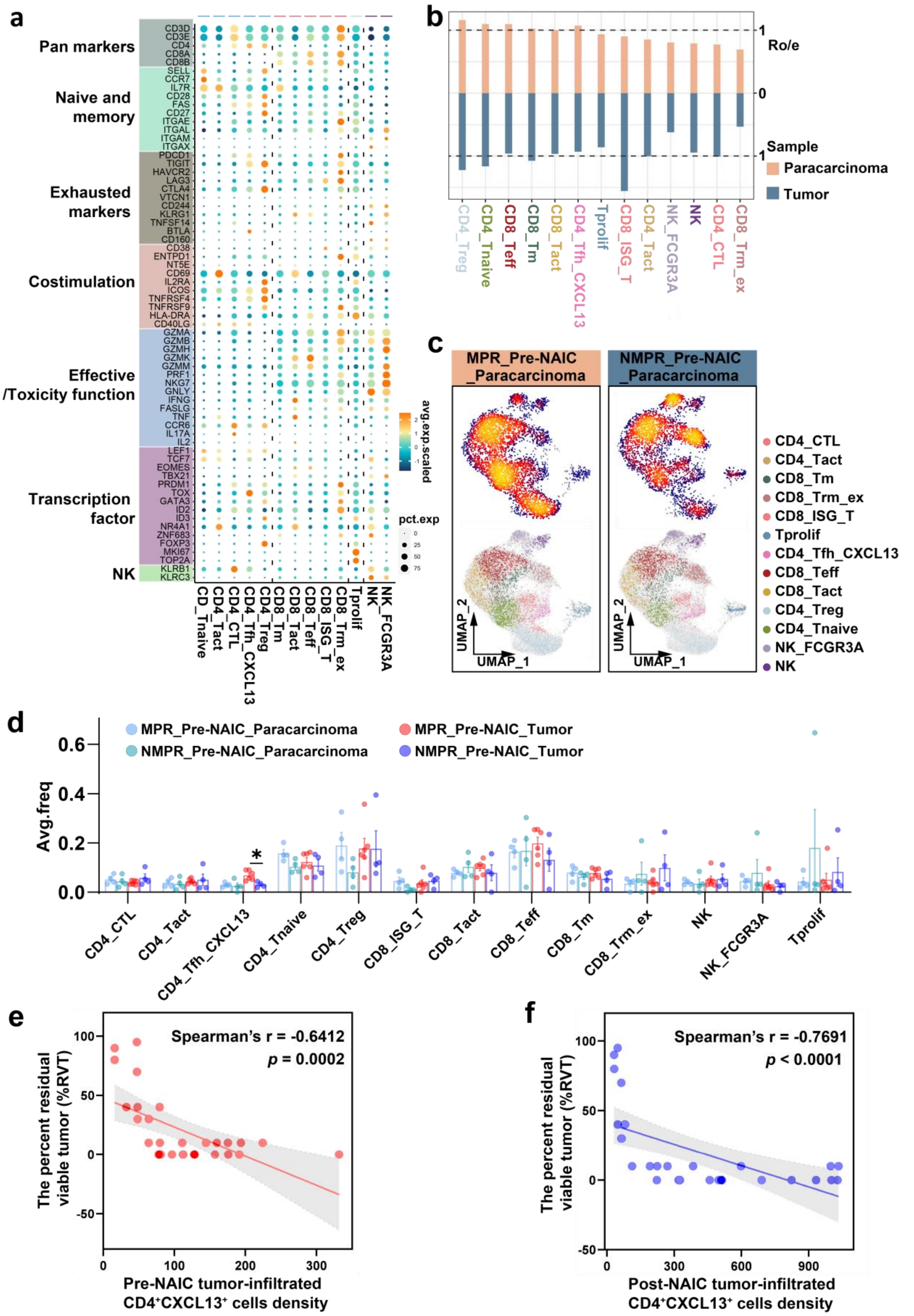

Supplementary Figure 5. Single-cell RNA-seq analysis in T/NK cells. a, Representative marker genes for each T/NK cell sub-cluster. b, Analysis of single-cell tissue preference for T cell sub-clusters in paracancerous and tumor tissues. c, UMAP illustrations of changes in the number of T cell subtypes between MPR and NMPR

groups in paracancerous tissue before NAIC. d, Quantification of all T/NK cell subtypes in paracancerous and tumor tissues before NAIC. Data are presented as mean values with SD. Two-sided unpaired t test was used for statistical analysis. e, Spearman's correlation analysis between pre-NAIC tumor-infiltrated CD4_Tfh_CXCL13 cells and %RVT (Spearman r = -0.6412, p = 0.0002). f, Spearman's correlation analysis between post-NAIC tumor-infiltrated CD4_Tfh_CXCL13 cells and %RVT (Spearman r = -0.7691, $p < 0.0001$). Gray area represent 95% confidence interval. Two-sided test was used for statistical analysis. %RVT, percent of residual viable tumor; MPR, major pathologic response; NMPR, non-major pathologic response; Ro/e, the ratio of observed over expected cell numbers. The dot represents an individual data point. Source data are presented as a Source Data file. * for $p < 0.05$.

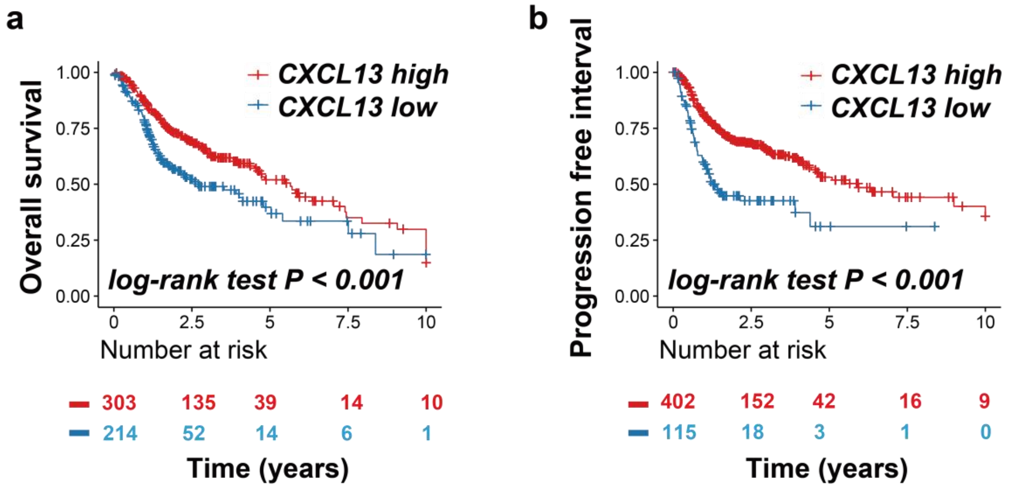

Supplementary Figure 6. Kaplan-Meier analyses of overall survival (p = 0.00076) (a) and progression free survival (p = 0.00038) (b) in patients with high versus low *CXCL13* expression in the TCGA head and neck cancer cohort. Log-rank test was used for statistical analysis.

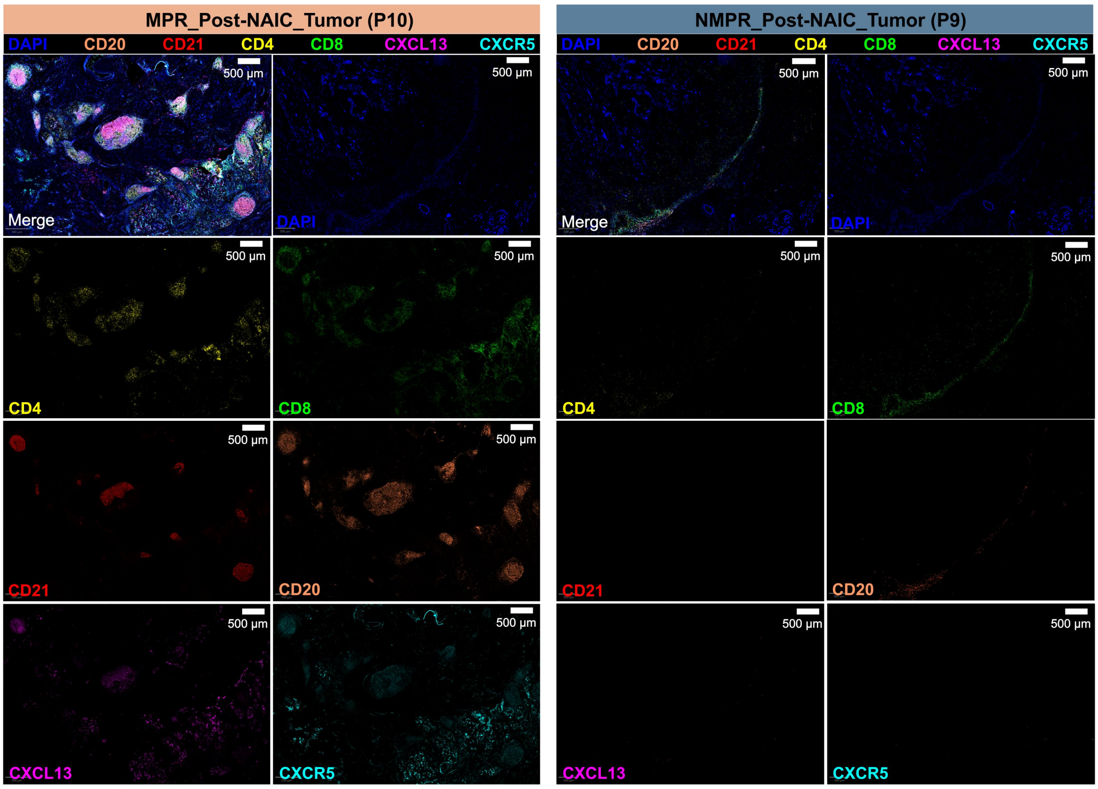

Supplementary Figure 7. Representative images (from patient P9 and P10) of mature phenotypes of tertiary lymphoid structures validated by multiplex immunofluorescence. NAIC, neoadjuvant immunochemotherapy; MPR, major pathologic response; NMPR, non-major pathologic response.

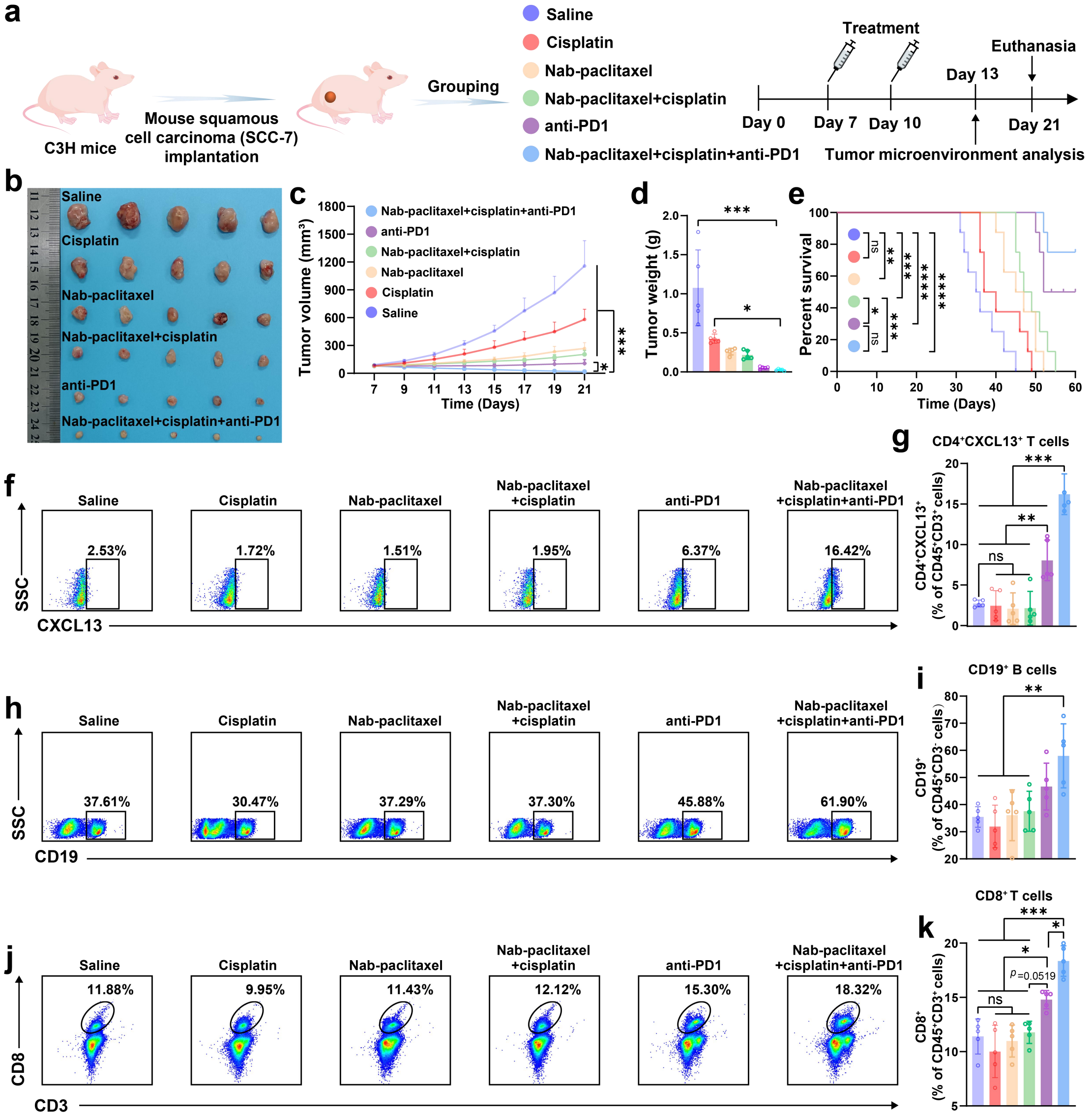

Supplementary Figure 8. Exploration of the immune function of each agent in the immunochemotherapy regimen *in vivo*. a, Schematic illustration of the experimental design (Created in Adobe Illustrator 2024). b, Representative tumors after 14 days of treatment. c, Tumor growth curves after different treatments (n = 5 in each group), data are presented as mean values with SD. Two-way ANOVA with Tukey's multiple comparisons test was used for statistical analysis. d, Tumor weights after 14 days of treatment (n = 5 in each group), data are presented as mean values with SD, the dot represents an individual data point. One-way ANOVA with Tukey's multiple comparisons test was used for statistical analysis. e, Survival curves of mice after different treatments (n = 5 in each group). Log-rank test was used for statistical

analysis. f and g, $CD4^{+}CXCL13^{+}$ T cell ratios in tumors after different treatments by flow cytometry analysis (n = 5 in each group). h and i, $CD3^{-}CD19^{+}$ B cell ratios in tumors after different treatments by flow cytometry analysis (n = 5 in each group). j and k, $CD3^{+}CD8^{+}$ T cell ratios in tumors after different treatments by flow cytometry analysis (n = 5 in each group). Data are presented as mean values with SD, the dot represents an individual data point. One-way ANOVA with Tukey's multiple comparisons test was used for statistical analysis. *ns* means no significant difference, * p <0.05, ** p <0.01, *** p <0.001. Source data and p values are presented as a Source Data file.

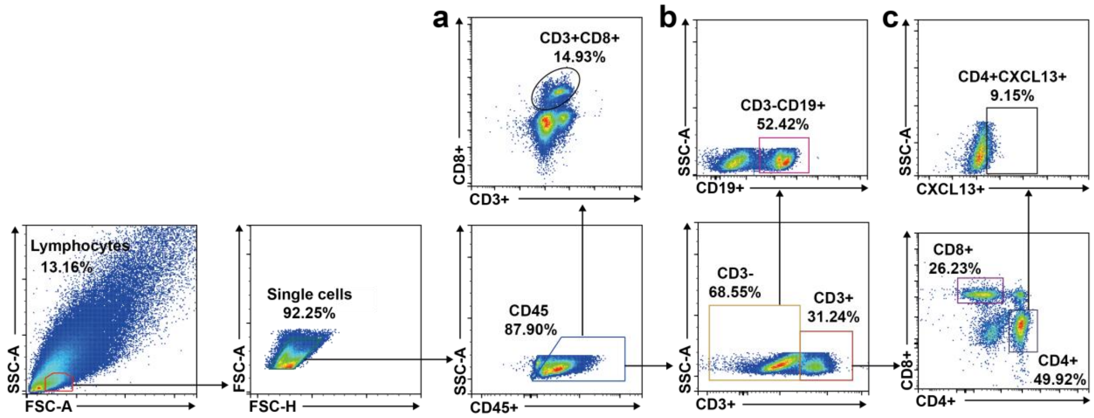

Supplementary Figure 9. Flow cytometry detection of tumor-infiltrated lymphocyte from C3H mice. a, Gating strategy for assessing the proportions of $CD3^{+}CD8^{+}$ T cells in tumor. b, Gating strategy for assessing the proportions of $CD3^{-}CD19^{+}$ B cells in tumor. c, Gating strategy for assessing the proportions of $CD4^{+}CXCL13^{+}$ T cells in tumor.

Supplementary Table 1. Clinical to pathological downstage after neoadjuvant immunochemotherapy

Patient number	Clinical stage (Pre-NAIC)	Clinical stage (Post-NAIC)	Pathological stage (Post-NAIC)	Residual viable tumor (%RVT)		Radiographic response (%)	Clinical to pathological downstage		Clinical downstage	
				Primary tumor	Lymph node		Primary tumor	Lymph node	Primary tumor	Lymph node
P1	cT3N0M0 (stage III)	cT1N0M0 (stage I)	ypT1N0M0 (stage I)	MPR (%RVT < 10%)	-	PR (-67.2%)	Yes	-	Yes	-
P2	cT4aN2cM0 (stage IVA)	cT4aN2cM0 (stage IVA)	ypT4aN2bM0 (stage IVA)	NMPR (%RVT = 90%)	NMPR (%RVT = 80%)	PR (-37.5%)	No	Yes	No	No
P3	cT4aN2cM0 (stage IVA)	cT1N1M0 (stage III)	ypT1N1M0 (stage III)	MPR (%RVT < 10%)	MPR (%RVT < 10%)	PR (-52.60%)	Yes	Yes	Yes	Yes
P4	cT3N2cM0 (stage IVA)	cT3N2cM0 (stage IVA)	ypT2N2cM0 (stage IVA)	NMPR (%RVT = 40%)	NMPR (%RVT = 50%)	SD (-29.83%)	Yes	No	No	No
P5	cT3N1M0 (stage III)	cT1N1M0 (stage III)	ypT1N1M0 (stage III)	NMPR (%RVT = 30%)	NMPR (%RVT = 60%)	PR (-64.50%)	Yes	No	Yes	No
P6	cT3N2bM0 (stage IVA)	cT3N2bM0 (stage IVA)	ypT3N2bM0 (stage IVA)	NMPR (%RVT = 70%)	NMPR (%RVT = 80%)	SD (-28.9%)	No	No	No	No
P7	cT3N1M0 (stage III)	cT1N0M0 (stage I)	ypT0N0M0 (-)	pCR (%RVT =	pCR (%RVT =	PR (-70.57%)	Yes	Yes	Yes	Yes

				0)	0)					
P8	cT3N1M0 (stage III)	cT3N1M0 (stage III)	ypT3N1M0 (stage III)	NMPR (%RVT = 80%)	NMPR (%RVT = 90)	SD (-9.96%)	No	No	No	No
P9	cT4aN2aM0 (stage IVA)	cT2N2aM0 (stage IVA)	ypT1N2aM0 (stage IVA)	NMPR (%RVT = 40%)	NMPR (%RVT = 70%)	PR (-51.72%)	Yes	No	Yes	No
P10	cT4aN3aM0 (Stage IVB)	cT2N1M0 (Stage III)	ypT1N0M0 (stage I)	MPR (%RVT< 10%)	pCR (%RVT = 0)	PR (-50.78%)	Yes	Yes	Yes	Yes
P11	cT4aN2aM0 (stage IVA)	cT2N1M0 (stage III)	ypT1N0M0 (stage IVA)	MPR (%RVT< 10%)	pCR (%RVT = 0)	PR (-57.55%)	Yes	Yes	Yes	Yes
P12	cT2N2aM0 (stage IVA)	cT2N2aM0 (stage IVA)	ypT1N2aM0 (stage IVA)	NMPR (%RVT = 40%)	NMPR (%RVT = 60%)	SD (-21.16%)	Yes	No	No	No
P13	cT3N2aM0 (stage IVA)	cT1N1M0 (stage III)	ypT1N2aM0 (stage IVA)	MPR (%RVT < 10%)	MPR (%RVT < 10%)	PR (-54.83%)	Yes	No	Yes	Yes
P14	cT3N2aM0 (stage IVA)	cT3N2aM0 (stage IVA)	ypT3N2aM0 (stage IVA)	NMPR (%RVT = 95%)	NMPR (%RVT = 80%)	PR (-35.11%)	No	No	No	No
P15	cT4aN0M0 (stage IVA)	cT2N0M0 (stage II)	ypT0N0M0 (-)	pCR (%RVT = 0)	-	PR (-43.43%)	Yes	-	Yes	-
P16	cT3N3bM0 (stage IVB)	cT1N2aM0 (stage IVA)	ypT0N1M0 (stage III)	pCR (%RVT = 0)	MPR (%RVT < 10%)	PR (-53.96%)	Yes	Yes	Yes	Yes
P17	cT4aN0M0	cT4aN0M0	ypT4aN0M0	NMPR	-	SD (-4.24%)	No	-	No	-

	(stage IVA)	(stage IVA)	(stage IVA)	(%RVT = 30%)						
P18	cT3N3bM0 (stage IVB)	cT1N3bM0 (stage IVB)	ypT1N3bM0 (stage IVB)	MPR (%RVT < 10%)	MPR (%RVT < 10%)	PR (-40.43%)	Yes	No	Yes	No
P19	cT3N2bM0 (stage IVA)	cT1N2bM0 (stage IVA)	ypT0N2bM0 (stage IVA)	pCR (%RVT = 0)	MPR (%RVT < 10%)	PR (-61.60%)	Yes	No	Yes	No
P20	cT3N1M0 (stage III)	cT1N0M0 (stage I)	ypT0N0M0 (-)	pCR (%RVT = 0)	pCR (%RVT = 0)	PR (-66.67%)	Yes	Yes	Yes	Yes
P21	cT3N1M0 (stage III)	cT1N0M0 (stage I)	ypT0N0M0 (-)	pCR (%RVT = 0)	pCR (%RVT = 0)	PR (-61.94%)	Yes	Yes	Yes	Yes
P22	cT3N2aM0 (stage IVA)	T0N0M0 (-)	ypT0N0M0 (-)	pCR (%RVT = 0)	pCR (%RVT = 0)	CR (-100%)	Yes	Yes	Yes	Yes
P23	cT4aN2bM0 (stage IVA)	T0N0M0 (-)	ypT0N0M0 (-)	pCR (%RVT = 0)	pCR (%RVT = 0)	CR (-100%)	Yes	Yes	Yes	Yes
P24	cT3N2aM0 (stage IVA)	T0N0M0 (-)	ypT0N0M0 (-)	pCR (%RVT = 0)	pCR (%RVT = 0)	CR (-100%)	Yes	Yes	Yes	Yes
P25	cT3N2aM0 (stage IVA)	T0N0M0 (-)	ypT0N0M0 (-)	pCR (%RVT = 0)	pCR (%RVT = 0)	CR (-100%)	Yes	Yes	Yes	Yes
P26	cT3N0M0 (stage III)	T0N0M0 (-)	ypT0N0M0 (-)	pCR (%RVT = 0)	-	CR (-100%)	Yes	-	Yes	-

P27	cT4aN2cM0 (stage IVA)	T0N0M0 (-)	ypT0N1M0 (stage III)	pCR (%RVT = 0)	MPR (%RVT < 10%)	CR (-100%)	Yes	Yes	Yes	Yes
P28	cT4aN2cM0 (stage IVA)	T1N0M0 (stage I)	ypT1N1M0 (stage III)	MPR (%RVT < 10%)	MPR (%RVT < 10%)	PR (-82.56%)	Yes	Yes	Yes	Yes
P29	cT3N1M0 (stage III)	T0N0M0 (-)	ypT1N1M0 (stage III)	MPR (%RVT < 10%)	MPR (%RVT < 10%)	CR (-100%)	Yes	No	Yes	Yes
P30	cT4bN1M0 (stage IVB)	-	-	-	-	-	-	-	-	-
P31	cT4bN2aM0 (stage IVB)	-	-	-	-	-	-	-	-	-

NAIC, neoadjuvant immunochemotherapy; MPR, major pathologic response; NMPR, non-major pathologic response; CPS, combined positive score; CR, complete response; PR, partial response; SD, stable disease; PD, progressive disease. %RVT, the percent of residual viable tumor. Note, P30 and P31 refused surgical resection and imaging examination for personal reasons after receiving 2 cycles of NAIC, thus the pathological stage, %RVT and radiographic response of these two patients could not be evaluate.

Supplementary Table 2. Neoadjuvant treatment related adverse events

Adverse events, n (%)	Grade 1	Grade 2	Grade 3	Grade 4	Any grade
Haematological					
Anaemia	1 (3.2)	0	0	0	1 (3.2)
Leucopenia	5 (16.1)	3 (9.7)	0	0	8 (25.8)
Thrombocytopenia	2 (6.5)	1 (3.2)	1 (3.2)	0	4 (12.9)
Neutropenia	5 (16.1)	2 (6.5)	0	1 (3.2)	8 (25.8)
Non-haematological					
Alopecia	31 (100)	0	0	0	31 (100)
Nausea	19 (61.3)	0	0	0	19 (61.3)
Vomiting	10 (32.3)	0	0	0	10 (32.3)
Hepatoxicity (Elevated sera ALT/AST)	3 (9.7)	0	0	0	3 (9.7)
Nephrotoxicity (Elevated sera creatinine)	1 (3.2)	0	0	0	1 (3.2)
Fatigue	6 (19.4)	0	0	0	6 (19.4)
Diarrhoea	1 (3.2)	0	0	0	1 (3.2)
Immune-related					
RCCEP	5 (16.1)	0	0	0	5 (16.1)
Hyperthyroidism	1 (3.2)	0	0	0	1 (3.2)
Hypothyroidism	1 (3.2)	0	0	0	1 (3.2)
Pneumonia	1 (3.2)	0	0	0	1 (3.2)

Data are n (%). RCCEP, reactive cutaneous capillary endothelial proliferation; ALT, glutamic pyruvic transaminase; AST, glutamic oxaloacetic transaminase

Supplementary Table 3. Surgery related adverse events

Adverse events	Grade 1	Grade 2	Grade 3	Grade 4
Subcutaneous exudation	1 (3.4%)	0	0	0
Postoperative bleeding	0	1 (3.4%)	0	0
Pain	4 (13.8%)	0	0	0
Swelling	3 (10.3%)	0	0	0
Limitation of mouth opening	2 (6.9%)	1 (3.4%)	0	0
Hematoma	1 (3.4%)	0	0	0

Data are n (%).

Supplementary Table 4. Adjuvant (chemo) radiotherapy related or maintenance immunotherapy related adverse events

**Adverse events**	**Grade 1**	**Grade 2**	**Grade 3**	**Grade 4**
Haematological				
Anaemia	1 (3.4%)	0	0	0
Leucopenia	1 (3.4%)	0	0	0
Neutropenia	1 (3.4%)	0	0	0
Non-haematological				
Stomatitis (mucositis)	11 (37.9%)	0	0	0
Dry mouth	1 (3.4%)	0	0	0
Dermatitis	3 (10.3%)	0	0	0
Dysphagia/odynophagia	2 (6.9%)	0	0	0
Nausea	1 (3.4%)	0	0	0
Immune-related				
RCCEP	1 (3.4%)	0	0	0
Hypothyroidism	1 (3.4%)	0	0	0

Data are n (%). RCCEP, reactive cutaneous capillary endothelial proliferation.

References

[1] Bankhead, P. et al. QuPath: Open source software for digital pathology image analysis. *Sci. Rep*. **4**, 16878 (2017).

[2] Barua, S. et al. Spatial interaction of tumor cells and regulatory T cells correlates with survival in non-small cell lung cancer. *Lung. Cancer*. **117**, 73-79 (2018).

Supplementary note: Study protocol

Neoadjuvant camrelizumab plus albumin paclitaxel and cisplatin in locally advanced oral squamous cell carcinoma: A phase II Clinical Trial

Version number/version date: Version 2.0/January 31, 2024

Trial sponsor: West China Hospital, Sichuan University

Statistical analyst division: West China Hospital, Sichuan University

Principal investigator: Lei Liu

Trial start time: January 30, 2023

Version/revision history

<table>
<tr><th>File</th><th>Version date</th><th>Explanation of the reason for the amendment and summary of the amendment</th></tr>
<tr><td>Version 1.0</td><td>October 31, 2022</td><td rowspan="2">The reason for the amendment: In the subsequent analysis stage, we added the exploratory outcome, bio-radiomics analysis.

The summary of the amendments:

“3. Exploratory Endpoints

1) Bio-radiomic markers analysis”

On page 1 in the part “II. Study Endpoints”.</td></tr>
<tr><td>Version 2.0</td><td>January 31, 2024</td></tr>
</table>

I. Summary

Tittle: Neoadjuvant camrelizumab plus albumin paclitaxel and cisplatin in locally advanced oral squamous cell carcinoma: A phase II Clinical Trial

Trial stage: Phase II

Sponsor: West China Hospital, Sichuan University

Responsible party: West China Hospital, Sichuan University

Principal investigator: Lei Liu

Research object: Locally advanced oral squamous cell carcinoma

Trial drugs: Camrelizumab (200 mg/vial), nab-paclitaxel (100 mg/vial), cisplatin (20 mg/vial)

Objective: To evaluate the efficacy and safety of camrelizumab combined with nab-paclitaxel/cisplatin neoadjuvant therapy in locally advanced oral squamous cell carcinoma.

Subjects: Patients with newly diagnosed locally advanced oral squamous cell carcinoma.

Sample size: 31 patients

Study endpoints:

1. **Primary endpoints**

    1) Major pathological response (MPR) rate: percentage of patients with residual visible tumor ≤ 10% of the total tumor bed area.

    2) Safety.

2. **Secondary endpoints**

1) Objective response rate (ORR).
2) Disease-free survival (DFS).
3) Overall survival (OS).
4) Correlation between pathological response and survival.
5) Pathological complete response (pCR) rate.

3. Exploratory endpoints

1) Bio-radiomic markers analysis.

Inclusion criteria:

1. Age: 18-75 years.
2. Histologically confirmed oral squamous cell carcinoma (including cancers of the tongue, gingiva, buccal mucosa, floor of mouth, and retromolar trigone).
3. Locally advanced oral squamous cell carcinoma (Stage III-IVB according to the 8th edition UICC/AJCC staging).
4. ECOG performance status 0-1.
5. No prior relevant treatment.
6. No distant metastasis.
7. Intention for curative treatment.
8. Adequate hematopoietic function (total white blood cell count ≥ 3.5×10^9 /L, absolute lymphocyte count ≥ 0.8×10^9 /L, absolute neutrophil count ≥ 1.5×10^9 /L, platelet count ≥ 100×10^9 /L, hemoglobin ≥ 90g/L).
9. Adequate liver function (bilirubin level ≤ 1.5 × upper limit of normal (ULN); AST and ALT levels ≤ 2.5 × ULN).
10. Adequate renal function (serum creatinine ≤ 1.5 × ULN or calculated creatinine clearance rate ≥ 60 ml/min by Cockcroft-Gault formula; urine protein ≤ 2+ on routine examination, or <1g in 24-hour urine protein quantification).

11. Adequate coagulation function, defined as international normalized ratio (INR) or prothrombin time (PT) $\leq 1.5 \times$ ULN; if the subject is receiving anticoagulant therapy, PT should be within the therapeutic range for the anticoagulant.

12. No significant organic heart disease or arrhythmias.

13. Women of childbearing potential (15-49 years) must have a negative pregnancy test within 7 days before starting treatment; sexually active male and female patients must agree to use effective contraception measures during the study and for 3 months after stopping treatment.

Exclusion criteria:

1. Patients with distant metastases.
2. Patients refusing surgical treatment.
3. Patients with palliative treatment intent.
4. Patients with prior treatment for primary or metastatic lymph nodes in any form of anti-tumor therapy, including chemotherapy, radiotherapy, targeted therapy, anti-PD-1 or PD-L1 therapy, or surgery (except biopsy).
5. Patients with other malignant tumors.
6. Active infections.
7. Patients with a history of organ transplantation.
8. Patients with a history of autoimmune diseases, or other diseases requiring long-term systemic use of steroid medications or immunosuppressive therapy.
9. Positive for human immunodeficiency virus (HIV).
10. Active hepatitis B or hepatitis C infection (HBV DNA, HCV RNA exceeding normal values).
11. Abnormal hematologic parameters: total white blood cell count $< 3.5 \times 10^9$ /L, absolute lymphocyte count $< 0.8 \times 10^9$ /L, absolute neutrophil count $< 1.5 \times 10^9$ /L, platelet count $< 100 \times 10^9$ /L, hemoglobin < 90 g/L; elevated bilirubin > 1.5 times the upper limit of normal (ULN), AST or ALT > 3 times ULN (5 times ULN if liver

metastasis), serum creatinine > 1.5 times ULN; abnormal coagulation function, international normalized ratio (INR) or prothrombin time (PT) > 1.5 times ULN.

12. Severe cardiovascular, respiratory, or immune system disorders, including urinary tract obstruction, myocardial infarction, arrhythmias, obstructive or restrictive pulmonary disease, or other conditions deemed by the investigator to increase participant risk.
13. Pregnant or lactating women.
14. Patients unwilling to use effective contraception during the study and for 3 months after stopping treatment.
15. Patients concurrently participating in other clinical studies.
16. Critically ill patients unable to complete the study assessments.
17. Patients with a history of psychiatric disorders (such as schizophrenia, bipolar disorder, anxiety disorders, depression, phobias, etc.) or those diagnosed with psychiatric disorders at enrollment, or their spouses.
18. Patients with communication barriers due to conditions like confusion, aphasia, intellectual disability, etc., affecting normal response.
19. Patients with allergies and congenital immune deficiencies.
20. Patients intolerant to immunotherapy.
21. Other factors as judged by the investigator that may deem the participant unsuitable or affect their participation or completion of the study.

Study design

1. **Study type:** Phase II, single-arm, exploratory study.
2. **Randomization:** Non-controlled setting.
3. **Study center:** West China Hospital of Sichuan University.

Sample size: Simon two-stage optimal design with an one-sided α value of 0.05 and a power of 80% to assess the efficacy of the new NAIC regimen of camrelizumab plus

nab-paclitaxel and cisplatin. We assumed that the new NAIC regimen could increase the MPR to 55% compared to a historical control of 27.7% with docetaxel 75 mg/m^2, cisplatin 75 mg/m^2, and fluorouracil 750 mg/m^2 (NCT01542931). In the first stage, 9 patients were required. If more than 3 patients achieved MPR, recruitment would proceed to the second stage with an additional 19 patients. The total sample size was up to 28 patients in this two-stage phase 2 study. Considering a dropout rate of 10%, a total of 31 patients were needed in the study. If more than 11 patients achieved MPR among 28 patients eligible for the MPR evaluation, this indicated that the new regimen was worth for further exploration.

Medical records/samples

1. **Medical records name:** This study plans to include newly diagnosed locally advanced oral cancer patients treated at the head and neck oncology department of West China Hospital, Sichuan University, numbered sequentially by enrollment.
2. **Medical records source:** Medical history, surgical records, and postoperative pathological data extracted from the hospital information system (HIS).
3. **Medical records period:** December 2022 - December 2025.
4. **Medical records acquisition**: Medical history, surgical records, postoperative pathology, and hematological data collected through inpatient observation, outpatient follow-up, or WeChat follow-up to monitor treatment-related adverse events.
5. **Handling and disposal of samples:** Biopsy and surgical pathology specimens are stored by West China Hospital for backup.

Criteria for stopping or terminating clinical research

1. **Investigator decision to withdraw patients:** Patient withdrawal refers to cases where enrolled patients encounter conditions during the study that make it inappropriate to continue treatment. The investigator considers the risk to the patient

participating in the study to be greater than the benefit, and decides to withdraw the case from the study.

1) Clear evidence of tumor progression during the study.

2) Occurrence of serious adverse events or other conditions making continuation of the study inappropriate.

3) Poor compliance with the treatment regimen resulting in inadequate use of therapeutic drugs.

4) Use of prohibited drugs or foods as specified in the protocol.

5) Use of other medications during the study that may affect efficacy, tolerance, and safety assessments.

6) Unexpected pregnancy in female patients during the study.

7) Other circumstances deemed necessary by the investigator to withdraw from the study, such as inability of the subject to express free will due to incarceration or isolation.

8) Loss to follow-up.

2. **Patient self-withdrawal**: Patients have the right to withdraw from the study midway but should inform the investigator of their decision and the reasons for withdrawal should be understood and recorded as much as possible.

1) Subject withdraws informed consent to participate in the study and refuses further follow-up.

2) Difficulty tolerating certain adverse reactions, which prevent further participation in clinical research, etc.

3. **Criteria for terminating research**

1) Patients who choose not to meet inclusion criteria, but meet exclusion criteria.

2) Non-performers.

3) No data after enrollment.

4) Before data analysis, statistical personnel and principal investigators discuss whether to exclude.

4. **Steps for withdrawing or terminating study treatment:** Efforts must be made to complete the validity and safety checks of the withdrawal or termination of study treatment specified in the plan, and complete safety follow-up, complete recording of adverse events (AEs) and outcomes. Researchers may suggest or provide new or alternative treatments based on the actual situation of the subjects. Non-disease progression subjects need to continue follow-up for radiological evaluation until subjects start new antitumor treatment or disease progression. If the subject refuses to make further visits to the research center, continue to track and collect research-related information, unless the subject withdraws from the disclosure of further information or agrees to continue to be contacted. In this case, no further evaluation should be performed, and no further data should be collected.

5. Early termination or suspension of the study: If justified, the study may be terminated or suspended early. This could be due to decisions from regulatory authorities, changes in ethical committee opinions, issues with the efficacy or safety of the study drug, or at the discretion of the sponsor. The party deciding to suspend/terminate the study will issue a written notification and record the reasons for terminating or suspending the study to the investigators, sponsor, and regulatory authorities. The investigator must immediately notify the ethics committee and the sponsor, providing relevant reasons.

Reasons for early termination or suspension of the study may include:

1) Identified unexpected, significant, or unacceptable risks to subjects.
2) Existing efficacy results support early termination of the study.
3) Low compliance as required by the protocol.

Once issues causing study suspension, such as drug safety, protocol compliance, etc., are resolved and agreed upon by the sponsor, ethics committee, or regulatory agency, the study may continue.

II. **Overview treatments**

1. Preoperative neoadjuvant therapy

Subjects meeting inclusion/exclusion criteria will first receive a treatment regimen of 2 cycles of camrelizumab (200 mg, Day 1, IV infusion) + nab-paclitaxel (260 mg/m^2, Day 1, IV infusion) + cisplatin (75 mg/m^2, divided into 3 days, Day 1-3, IV infusion) every 3 weeks.

2. Surgical treatment

Surgery will be performed 2-4 weeks after completion of neoadjuvant therapy. Depending on the extent of tumor invasion into tissue, radical resection of the primary lesion or neck lymph node dissection will be chosen, with appropriate use of pedicle or free flap for postoperative defect repair.

3. Postoperative adjuvant therapy

After comprehensive assessment of patient age, comorbid conditions, postoperative pathological staging, surgical margin status, extent of lymph node involvement, and other histopathological features of the primary tumor, a decision will be made whether to administer postoperative adjuvant radiotherapy or chemoradiotherapy (based on specific risk factors: for high-risk patients postoperatively, such as stage III-IV, close margins, tumor infiltration depth > 10 mm, vascular cancer embolism, neural invasion, positive lymph nodes, simple radiotherapy will be performed, and for patients with positive surgical margins or extracapsular invasion of lymph nodes, concurrent chemoradiotherapy based on cisplatin will be

performed). Additionally, patients will receive 6 cycles of camrelizumab (200 mg, IV infusion, every 3 weeks) for maintenance therapy.

Radiotherapy was performed within 6 weeks after surgery. The recommended radiation dose was 54-60 Gy (1.8-2.0 Gy perday, 5 days perweek, for 6 weeks). In patients with positive resectional margin and/or extranodal extension, cisplantin-based concurrent chemoradiotherapy was recommended, and a total of 66 Gy was allowed. A 20% dose reduction of cisplantin was required for patients experiencing grade 3 neutropenia, grade 2 thrombocytopenia, creatinine clearance of 40–60 mL/min, or grade 2 neurotoxicity in concurrent chemoradiotherapy phase. Radiotherapy was suspended for grade 4 oral mucositis, radiodermatitis, or radiation-induced myelosuppression.

III. **Study procedures**

Screening period

1. After subjects are fully informed about the study, they voluntarily sign the informed consent form. Screening procedures specified by the study can only proceed after signing the informed consent form.
2. Assign subject identification numbers.
3. Collect demographic information, medical history, treatment history, etc., from subjects.
4. Measure vital signs of subjects.
5. Complete the following examinations: pathological biopsy of lesion tissue, complete blood count (CBC), blood chemistry, coagulation profile, thyroid function panel, CK-MB, BNP, troponin, blood glucose or HbA1c, routine urine and stool tests, electrocardiogram (ECG), echocardiography, HIV antibody testing, hepatitis B (HBsAg, HBsAb, HBcAb, HBeAg, HBeAb) and HBV-DNA testing, and HCV antibody testing, pregnancy testing for non-menopausal females.

6. Assess examination results to verify inclusion/exclusion criteria and determine subject eligibility for the study.

7. Schedule admission dates for subjects eligible for neoadjuvant therapy.

8. Perform comprehensive nutritional status assessment for all subjects. Provide nutritional intervention promptly for patients at risk of malnutrition or nutritional deficiency to ensure smooth treatment implementation; others receive nutritional guidance. Advise current smokers to quit and counsel former smokers to avoid smoking again.

Pre-treatment assessment of primary lesion

Subjects undergo enhanced MRI of the oral and maxillofacial region, enhanced MRI of the neck, plain CT of the head and neck, abdominal ultrasound, and whole-body bone scintigraphy for radiological examinations to assess the primary lesion. Record size and location of each primary lesion according to Response Evaluation Criteria in Solid Tumors version 1.1 (RECIST 1.1) criteria.

Neoadjuvant therapy phase

1. Inquiring about recent adverse events, new concurrent medications, etc.
2. Measuring vital signs (including temperature, blood pressure, heart rate, respiratory rate, taken after sitting quietly for 5 minutes), perform physical examination, ECOG PS assessment, quality of life questionnaire, and record height, weight, etc.
3. Completing the following examinations: CBC, blood chemistry, coagulation profile, thyroid function panel, CK-MB, BNP, troponin, blood glucose or HbA1c, routine urine and stool tests, ECG. Assess subject condition based on examination results to determine suitability for continued neoadjuvant therapy.
4. Administering intravenous infusion of camrelizumab (200 mg) + nab-paclitaxel formulation (260 mg/m^2) on day 0 and day 21, and cisplatin (75 mg/m^2, divided into 3 days) on day 1-3 and day 22-24, totaling 2 cycles of neoadjuvant therapy.

5. Monitoring subjects closely during infusion for adverse reactions. Manage promptly if adverse reactions occur and maintain records.

6. Instructing subjects to return to the hospital on time for the next treatment/follow-up.

7. During the follow-up period after treatment, conduct regular CBC, blood chemistry, coagulation profile, thyroid function panel, CK-MB, BNP, and troponin to monitor and evaluate adverse reactions.

8. Evaluating occurrence of adverse reactions at the end of each cycle. If no severe adverse reactions occur, subjects may proceed with the next cycle of treatment.

9. After neoadjuvant: performing enhanced MRI of the oral and maxillofacial region, enhanced MRI of the neck, plain CT of the chest, abdominal ultrasound to evaluate response of primary lesions to neoadjuvant therapy based on RECIST 1.1 criteria.

RECIST 1.1 evaluation criteria:

1) Complete response (CR): Except for nodular diseases, all target lesions completely disappear. All target nodules must be reduced to normal size (short axis < 10 mm). All target lesions must be evaluated.

2) Partial response (PR): The total diameter of all measurable target lesions is 30% or lower than baseline. The sum of target nodules uses the short diameter, while the sum of all other target lesions uses the longest diameter. All target lesions must be evaluated.

3) Progressed disease (PD): Using the minimum sum of the diameters of all measured target lesions throughout the entire experimental study as a reference, the relative increase in diameter should be at least 20% (if the baseline measurement is the smallest, the baseline value should be used as a reference); in addition, it is necessary to increase the absolute value of the diameter by at least 5 mm (the appearance of one or more new lesions is also considered disease progression).

4) Stable disease (SD): The degree of reduction of the target lesion has not reached PR, and the degree of increase has not reached PD level, which is between the two. The minimum value of the sum of diameters can be used as a reference for research.

Surgical period

1. Inquiring about recent adverse events, new concurrent medications, etc.
2. Measuring vital signs (including temperature, blood pressure, heart rate, respiratory rate, taken after sitting quietly for 5 minutes), perform physical examination, ECOG PS assessment, quality of life questionnaire, and record height, weight, etc.
3. Completing the following examinations: CBC, blood chemistry, coagulation profile, pre-transfusion tests, thyroid function panel, CK-MB, BNP, troponin, blood glucose or HbA1c, routine urine and stool tests, ECG, echocardiography. Assess subject condition based on examination results to determine suitability for surgery.
4. Selecting definitive surgery for primary lesion or cervical lymph node dissection based on extent of tumor tissue involvement. Use pedicle or free flaps as needed for postoperative defect repair.
5. Monitoring subjects closely perioperatively for vital signs and monitor surgical area for bleeding, wound dehiscence, infection, etc. Manage promptly if abnormalities occur and maintain records.
6. Instructing subjects to return weekly for follow-up visits. Observe for abnormal changes in surgical area and conduct regular CBC, blood chemistry, coagulation profile, thyroid function panel, CK-MB, BNP, troponin to monitor and evaluate adverse reactions.

Postoperative adjuvant radiotherapy period

1. Waiting for complete healing of surgical wounds before initiating radiotherapy.
2. Measure vital signs (temperature, blood pressure, heart rate, respiratory rate, taken after sitting quietly for 5 minutes), perform physical examination, ECOG PS

assessment, quality of life questionnaire, and record height, weight, etc. before radiotherapy.

3. Complete the following examinations before radiotherapy: CBC, blood chemistry, coagulation profile. Assess subject eligibility for radiotherapy based on examination results evaluated by radiation oncologists.

4. Preparing for localization

Oral care: Examining teeth, periodontium, and mucosa conditions. Understanding patient’s oral health methods, habits, outcomes, and awareness of oral diseases. Providing detailed oral hygiene guidance based on patient’s oral condition. Performing full-mouth dental cleaning. Treating gingivitis and chronic periodontitis. Managing and fill cavities, polishing sharp rough tooth tips, removing poor restorations. Extracting residual crowns/roots as needed cautiously to ensure accurate CT or MRI imaging without metallic teeth or filling materials affecting image quality. Carefully extracting teeth around lesions. Preparing oral radiotherapy stents: During radiation therapy for oral cancer, due to the complex anatomy and mobility of organs (such as tongue, cheek, mandible, etc.), an oral radiotherapy stent is required for fixation to ensure accuracy of target area during each radiotherapy session and avoid excessive radiation dose to surrounding normal organs, thereby reducing radiation-related adverse reactions such as oral mucositis, xerostomia, and taste disorders.

5. Localization: Reading imaging pictures carefully before localization to define tumor scope. Positioning the patient supine with a pillow, heat-molded head-neck-shoulder immobilization device, and oral radiotherapy stent. Perform enhanced CT scan for localization using CT/MRI image fusion technology. The range includes from the top of the skull to 2 cm below the clavicle (up to tracheal bifurcation level if necessary), with a scan slice thickness of 3 mm. Radiation oncologists outline target areas based on CT/MRI fusion localization images and develop radiotherapy prescriptions. Physicists create radiotherapy plans. Evaluate

feasibility of radiotherapy plan by radiation oncologists. Administer radiotherapy to subjects once daily, five times a week, completed in approximately 6 weeks. Conduct weekly follow-up visits during radiotherapy to observe oral mucosa and skin reactions in the irradiated area. Check CBC, blood chemistry. Manage promptly and consider suspending radiotherapy if radiation-related adverse reactions occur.

Maintenance therapy period

1. Continuing intravenous infusion of camrelizumab (200 mg) every 3 weeks for 6 cycles postoperatively.
2. Checking CBC, blood chemistry, coagulation profile, thyroid function panel, CK-MB, BNP, troponin, blood glucose or HbA1c, routine urine and stool tests, ECG, echocardiography before each infusion to evaluate subject's suitability for continuing camrelizumab maintenance therapy.
3. Evaluating occurrence of adverse reactions at the end of each cycle. If no severe adverse reactions occur, subjects may proceed with the next cycle of treatment.
4. Instructing subjects to return weekly for follow-up visits. Observe for abnormal changes in surgical and irradiated areas and conduct regular CBC, blood chemistry, coagulation profile, thyroid function panel, CK-MB, BNP, troponin to monitor and evaluate adverse reactions.

Follow-up period

At the end of radiotherapy or upon decision to terminate the study, the follow-up period begins. Subjects return for their first follow-up visit one month after completion of radiotherapy. Examination includes CBC, blood chemistry, coagulation profile, thyroid function panel, CK-MB, BNP, troponin, routine urine and stool tests, ECG, echocardiography, and physical examination. Follow-up visits occur every 3 months for 2 years, every 6 months in the 3-5 years, and annually thereafter.

IV. **Optional alternative diagnosis and treatment methods**

The treatment regimen of camrelizumab combined with nab-paclitaxel/cisplatin for neoadjuvant treatment of locally advanced oral squamous cell carcinoma is still under exploration in this study. Subjects who withdraw from clinical research before or during treatment may still choose to undergo surgery ± adjuvant radiotherapy/chemotherapy or other treatment regimens.

V. Efficacy evaluation criteria

For detailed evaluation criteria, please see previous content.

VI. Investigational medications

Information on Study Drugs

1. **Camrelizumab**
    - Generic name: Injection camrelizumab
    - English name: Camrelizumab for Injection
    - Manufacturer: Hengrui Pharmaceuticals Co. Ltd, China.
    - Dosage form: Injection
    - Specification: 200 mg/vial
    - Administration: Intravenous injection
    - Shelf life: 24 months from production date
    - Storage: Store and transport between 2-8°C, protect from light
2. **Nab-paclitaxel**
    - Generic name: Injection paclitaxel (Albumin Bound)
    - Manufacturer: Hengrui Pharmaceuticals Co. Ltd, China.

- Dosage form: Lyophilized powder
- Specification: 100 mg/vial
- Administration: Intravenous injection
- Shelf life: 12 months from production date
- Storage: Protect from light, store at 20-30°C

3. **Cisplatin**
    - Generic name: Injection cisplatin
    - Manufacturer: Jiangsu Hansoh Pharmaceutical Group Co., Ltd.
    - Dosage form: Injection
    - Specification: 20 mg/vial
    - Administration: Intravenous injection
    - Shelf Life: 12 months from production date
    - Storage: Protect from light, store in a tightly closed container

Drug management

After subjects signing the informed consent form and complete necessary baseline assessments, they will automatically be assigned a subject ID, which will be used for all CRFs and study documents. After completing the screening process, subjects are formally enrolled in the study. The study center staff will send a complete Subject Enrollment Assessment Form (Eligibility Form) to the study team members designated by the sponsor via email.

No subject will receive study drugs until the investigator or designee receives the following written information:

- Confirmation of subject enrollment
- Clarification of the subject’s dose level
- Permission to administer the subject

Preparation and dispense of drugs

The management, distribution, and retrieval of investigational drugs in this study are managed by designated personnel. Researchers must ensure that all investigational drugs are used only for subjects participating in the clinical trial, dosages and usage should follow the trial protocol. Clinical drugs must not be transferred to any non-clinical trial participants. When distributing drugs to research centers, a drug receipt must be signed by two people, with two copies. At the end of the study, the remaining drugs and empty boxes will be recovered, and a drug recovery form will be signed by both parties. Each drug distribution and recovery should be promptly recorded on a dedicated record sheet.

Administration

- Camrelizumab 200 mg, ivgtt. Day 1, once every three weeks for 2 cycles;
- nab-paclitaxel 260 mg/m^2, ivgtt. Day 1, once every three weeks for 2 cycles;
- Cisplatin 75 mg/m^2, ivgtt. Divided into 3 days, Day 1-3, once every three weeks for 2 cycles.

Drug dose adjustment

For grade 3 adverse events such as capillary proliferative reaction, platelet reduction syndrome, neutropenia, anemia, grade 2 pneumonia, hepatitis, myocarditis, adrenal insufficiency, grade 2-3 diarrhea, nephritis, hypothyroidism, hyperthyroidism, and pancreatitis, Camrelizumab administration should be temporarily suspended. Treatment may resume when these adverse events recover to ≤ grade 1. Patients with grade 3-4 pneumonia, hepatitis, adrenal insufficiency, myocarditis, grade 4 hypothyroidism, hyperthyroidism, capillary proliferative reaction, diarrhea, or nephritis should permanently discontinue Camrelizumab. If patients experience grade 3-4 diarrhea, hepatitis, nephritis, platelet reduction syndrome, neutropenia, or anemia, nab-paclitaxel and cisplatin should be temporarily suspended until adverse events recover to ≤ grade 2, and subsequent drug doses should be reduced by 25%-50%.

Other anti-tumor/anti-cancer or investigational drugs

All treatments and medications used within 28 days prior to signing the informed consent form and during the study process should be strictly recorded in accordance with GCP regulations in the CRF. Subjects experiencing adverse events should be closely monitored and, if necessary, actively treated symptomatically, with medications used documented and explained on the CRF.

1. Drugs and treatments prohibited for all subjects during the study period

1) Subjects are prohibited from using CFDA-approved modern Chinese medicine preparations for treating head and neck squamous cell carcinoma indications (including but not limited to Deleisure Injection, Kanglite Injection, Edi Injection, Huai'er Granules, and Liver Compactor Tablets) and immunomodulators (including but not limited to interferons, interleukin-2, thymosin, etc.) during the study period.

2) During this study period, subjects are not allowed to simultaneously receive immunosuppressive therapy (except for managing drug-related adverse events).

3) During this study period, subjects are not allowed to use other investigational drugs for anti-tumor therapy.

2. Drugs and treatments to be used with caution by all subjects during the study period

1) Drugs that interfere with hepatic P450 enzymes:

- **CYP3A4 inducers:** Dexamethasone, phenytoin, carbamazepine, rifampicin, phenobarbital, levofloxacin, etc.
- **CYP3A4 inhibitors:** Itraconazole, clarithromycin, voriconazole, telithromycin, saquinavir, etc.
- **Drugs metabolized by CYP3A4:** Such as benzodiazepines, dihydropyridines, calcium channel blockers like nifedipine and amlodipine, etc.
- **HMG-CoA reductase inhibitors:** Simvastatin, midazolam, etc.
- **Drugs metabolized by CYP2C9:** Warfarin, phenytoin, certain sulfonylureas like glipizide, etc.

2) Drugs that prolong the QT interval:

- Including antibiotics, antiarrhythmic drugs, antipsychotic drugs, antifungal drugs, antimalarial drugs, antidepressants, etc.

3. **Drugs and treatments permitted to be used concurrently during the study period**

- **Corticosteroids:** 1) Local application of corticosteroids such as topical, ocular, nasal, intra-articular, and inhalational use is permitted; 2) corticosteroid replacement therapy for adrenal insufficiency is permitte; 3) corticosteroids for managing adverse reactions are permitted; 4) short-term use for preventing and treating allergic reactions (e.g., contrast media allergy or other allergies) is permitted.
- **Other Systemic Treatments:** During treatment, subjects should receive optimal supportive care. Use of pre-existing steroid replacement therapy is permitted.

VII. Supportive treatment

Supportive and palliative treatment for disease-related symptoms will be at the discretion of the investigator and in accordance with relevant guidelines (such as the American Society of Clinical Oncology (ASCO) guidelines).

VIII. Safety assessment

1. Adverse events (AEs)

1) Definition of AEs

- AEs refer to any untoward medical occurrence in a clinical trial subject who has received study treatment, from the start of treatment until 30 days after the last administration of the investigational drug. This includes unfavorable signs, symptoms, laboratory findings, or diseases, regardless of whether they are related to the investigational drug. AEs include:
    - Aggravation of pre-existing (prior to clinical trial entry) medical conditions/diseases (including worsening symptoms, signs, and worsening of laboratory abnormalities).
    - Newly occurring AEs: Any newly occurring unfavorable medical condition (including symptoms, signs, and newly diagnosed diseases).
    - Clinically significant abnormal laboratory values or results.
- Researchers should meticulously record all AEs experienced by subjects, including the AE name, description of all relevant symptoms, onset time, severity, relationship to the investigational drug, duration, actions taken, and final outcome and resolution.

2. Severity assessment criteria for AEs

Severity grading should follow the NCI-CTCAE (version 5.0) grading criteria for drug-related AEs. For AEs not listed in NCI-CTCAE (version 5.0), refer to the following criteria:

Grade 1: Mild; asymptomatic or mild symptoms; clinical or laboratory finding only; no intervention required.

Grade 2: Moderate; minimal, local, or non-invasive intervention indicated; limiting age-appropriate instrumental activities of daily living (ADL), such as cooking, shopping, telephoning, handling money, etc.

Grade 3: Severe or medically significant but not immediately life-threatening; hospitalization or prolongation of hospitalization indicated; disabling; limiting

self-care ADL. Self-care ADL includes bathing, dressing and undressing, feeding oneself, using the toilet, taking medications, not confined to bed.

Grade 4: Life-threatening consequences; urgent intervention indicated.

Grade 5: Death related to AE.

3. Assessment criteria for relationship between AE and investigational drug

During treatment, any discomfort reported by the subjects or objective abnormal changes in laboratory indicators should be accurately recorded. The severity, duration, management measures, and outcome of AE should be noted. The investigator should comprehensively assess the relationship between AE and the investigational drug using a five-level classification: "Definitely Related," "Probably Related," "Possibly Unrelated," "Definitely Unrelated," and "Uncertain."

4. Criteria for judging relationship between AE and investigational drugs

The criteria for determining the relationship between AEs and treatment can be evaluated according to the following 5-level classification criteria:

1) **Definitely related:** Clinical events, including laboratory abnormalities, have a reasonable sequential relationship with the use of the investigational drug and cannot be explained by accompanying diseases or other drugs. The discontinuation reaction is clinically reasonable. The event must be positively correlated with the pharmacological or clinical manifestations of the investigational drug and reappear upon re administration.

2) **Most likely related:** clinical events include laboratory abnormalities, which have a reasonable sequential relationship with the use of the investigational drug and are unlikely to be caused by accompanying diseases or other drugs. The discontinuation reaction is clinically reasonable. When administered again, this event may not necessarily occur.

3) **May be related:** Some clinical events, including laboratory abnormalities, have a reasonable sequential relationship with the use of the investigational drug, but can also be explained by accompanying diseases or other drugs. Discontinuation information may be missing or unclear.

4) **May be unrelated:** A clinical event that includes laboratory abnormalities may have a sequential relationship with the use of the investigational drug, but may not have a causal relationship with the investigational drug. Other drugs or diseases can provide reasonable explanations for causal relationships.

5) **Unevaluable:** Adverse events that do not meet any of the above criteria.

5. Serious adverse events (SAE)

1) Definition of SAE

SAE refers to medical events occurring during a clinical trial (from the start of treatment) that result in hospitalization or prolongation of hospitalization, disability, impairment of work ability, life-threatening conditions, death, or congenital anomalies. It includes the following unexpected medical events:

- Events resulting in death.
- Life-threatening events (defined as an event where there is an immediate risk of death if medical intervention is not undertaken at the time of the event, rather than assuming deterioration could lead to death).
- Events requiring hospitalization or prolongation of hospitalization.
- Events causing permanent or severe disability, functional impairment, or affecting work ability.
- Congenital anomalies or birth defects.
- Other significant medical events (defined as events that harm the subject or require intervention to prevent any of the above).

2) Hospitalization

Adverse events leading to hospitalization or prolongation of hospitalization during clinical research should be considered serious adverse events, excluding non-medical hospitalizations. Hospitalization or prolonged hospitalization not related to the worsening of adverse events (such as admission for pre-existing conditions, or management reasons) should not be reported as serious adverse events. For example:

- Admission due to pre-existing diseases without new adverse events occurring or exacerbation of pre-existing conditions (e.g., persistent laboratory abnormalities since before the trial).
- Management-related hospitalization (e.g., annual routine physical examination, hospitalization due to lack of accommodation, hospitalization for medical insurance reimbursement reasons).
- Hospitalization as per trial protocol requirements (e.g., operations as required by the trial protocol).
- Scheduled treatment or surgery should be recorded in the entire trial protocol and/or baseline data of the subject.

Diagnostic or therapeutic invasive (e.g., surgical) and non-invasive operations should not be reported as adverse events. However, if the disease condition leading to such operations meets the definition of an adverse event, it should be reported. For example, acute appendicitis occurring during the adverse event reporting period should be reported as an adverse event. The appendectomy performed as a result of this should be recorded as the treatment method for this adverse event.

3) **Reporting system for SAE**

The reporting period for SAEs extends from the start of treatment of the subject to 90 calendar days (including 90 days) after the last use of the investigational drug. If an SAE occurs, whether it is the first report or a follow-up report, the investigator must immediately complete the "Serious Adverse Event Report Form for New Drug Clinical Trials," sign and date it, and report it to the provincial, autonomous region,

municipality directly under the Central Government drug regulatory authority, CFDA, and the health administrative department (fax to the medical administration bureau) within 24 hours of the investigator's knowledge.

SAE occurring more than 90 days after the last use of the investigational drug, if suspected to be related to the investigational drug, also need to be reported.

SAE should be detailed with symptoms, severity, relationship with the investigational drug, occurrence time, handling time, measures taken, follow-up time and method, and outcome. If the investigator believes that an SAE is unrelated to the investigational drug but potentially related to study conditions (such as termination of original treatment or complications during the trial), this relationship should be detailed in the narrative section of the SAE report form.

If there is a change in the intensity of an ongoing SAE or its relationship with the investigational drug, a follow-up report should be submitted immediately.

4) **Follow-up of adverse events**

All adverse events should be followed up until recovery, resolution to baseline levels, ≤ Grade 1, or stabilization of the condition. If the event cannot recover to baseline levels or stabilize, a reasonable explanation should be recorded in the case report form (e.g., lost to follow-up, death, etc.).

All SAEs, regardless of their relationship with the investigational drug, need to be properly managed and the outcome of SAE recorded in the case report form along with the date.

The investigator should promptly provide follow-up information as per the requirements of the sponsor.

6. Pregnancy

If a female subject becomes pregnant during the clinical trial, the subject should be discontinued from the study. The investigator should report the pregnancy to the sponsor within 24 hours of knowledge and fill out the “Clinical Trial Pregnancy Report/Follow-up Form”.

If the partner of a male subject becomes pregnant during the clinical trial, the subject can continue in the trial. The investigator should report the partner's pregnancy to the sponsor within 24 hours of knowledge and fill out the “Clinical Trial Pregnancy Report/Follow-up Form”.

The investigator should follow up on the pregnancy outcome until 1 month after delivery and report the results to the sponsor.

If the pregnancy results in stillbirth, spontaneous abortion, fetal malformation, it is considered an SAE and needs to be reported according to the SAE reporting deadline requirements.

If a subject experiences an SAE during pregnancy, the “CFDA Serious Adverse Event Report Form” should also be completed, and the SAE reporting procedures should be followed.

7. Special attention AE

Special attention adverse events specified in the clinical trial protocol must be reported by the investigator within 24 hours of knowledge using the “Clinical Trial Special Attention Adverse Event Report Form” and reported to the sponsor. If the event is also an SAE, the “CFDA Serious Adverse Event Report Form: should be completed simultaneously.

List of special attention AEs:

- ≥ Grade 3 reactive cutaneous capillary endothelial proliferation
- ≥ Grade 3 infusion reaction

- ≥ Grade 2 diarrhea/colitis, uveitis, interstitial pneumonia
- ≥ Grade 3 other immune-related adverse events
- Any potential liver enzyme abnormality event (lacking other relevant causes such as disease progression, acute viral hepatitis, cholestasis, concurrent medication, pre-existing liver disease, etc.)
- Grade 4 [ipase or amylase elevation

8. Reactive cutaneous capillary endothelial proliferation

Reactive cutaneous capillary endothelial proliferation mostly occurs on the skin surface, with occasional cases in oral mucosa, nasal mucosa, and eyelid conjunctiva. Skin-reactive capillary proliferation initially appears as bright red pinpoint lesions on the skin. With repeated drug use, the lesions can gradually enlarge, presenting as nodular or patchy, bright red or dark red in color. Clinical symptoms and signs should be observed. Treatment should be based on the following grading criteria and treatment recommendations:

Grade 1: Single lesion with maximum diameter <10 mm, with or without ulceration or bleeding. Continue medication; protect areas prone to friction with gauze to prevent bleeding. For lesions with bleeding, apply local pressure for hemostasis if necessary.

Grade 2: Single lesion with maximum diameter >10 mm, with or without ulceration or bleeding. Continue medication; protect areas prone to friction with gauze to prevent bleeding. For lesions with bleeding, apply local pressure for hemostasis or consider local treatments such as laser therapy or surgical excision. Avoid infection at ulcerated sites.

Grade 3: Widespread lesions, may be complicated by skin infection, may require hospitalization. Discontinue medication temporarily; resume treatment after recovery to Grade 2. Protect areas prone to friction with gauze to prevent bleeding. For lesions with bleeding, apply local pressure for hemostasis or consider local

treatments such as laser therapy or surgical excision. Treat concurrent infections with appropriate antibiotics.

Grade 4: Multiple and widespread lesions, life-threatening. Discontinue medication permanently and seek immediate medical attention.

Grade 5: Death

IX. Data analysis

1. Evaluable analysis set (EAS): Participants who received at least one cycle of neoadjuvant treatment post-randomization, underwent at least one baseline imaging assessment, and had evaluable primary endpoint assessment data are included in this set. Cases missing baseline data for primary endpoint assessment are excluded from the FAS; ORR is analyzed within this set.

2. Per protocol set (PPS): Based on the FAS, this set includes cases that meet inclusion and exclusion criteria, have valid baseline values, primary efficacy endpoint assessments post-treatment, good compliance, and no major protocol deviations (e.g., use of prohibited drugs).

3. Surgery analysis set (SAS): Participants who received at least one cycle of neoadjuvant treatment post-randomization and underwent surgical treatment are included in this set; MPR and pCR are analyzed within this set.

4. Safety set (SS): Cases that received at least one study drug administration post-randomization are included.

5. Safety data: Detailed description of adverse event cases, calculation of incidence rates for different events.

X. Statistical analysis

1. Statistical methods primarily involve descriptive statistics. For continuous data, mean, standard deviation, maximum, and minimum values will be presented; for categorical and ordinal data, frequencies and rates will be listed. Statistical analyses will be conducted using GraphPad Prism (version 8.0.2) statistical software.

2. In all scatter plots and box plots, the median line, upper and lower boundaries represent the mean, 25th, and 75th percentiles, respectively. MPR, pCR, ORR, and AE will be presented as frequencies and percentages. Spearman correlation coefficient will assess correlations between potential biomarkers and pathological responses. Kaplan-Meier method will compute OS, DFS, and respective 95% confidence intervals, with survival curves plotted.

3. Safety Analysis:

1) All subjects will be monitored for adverse events during the clinical study, including clinical symptoms, vital sign abnormalities, and laboratory test anomalies. The characteristics, severity, onset time, duration, management, and prognosis of adverse events will be documented, and their relationship to the investigational drug assessed according to NCI-CTCAE (version 5.0) standards. For events within the same System Organ Class (SOC) and/or Preferred Term (PT), only the first occurrence per subject will be counted. In cases where the same adverse event is reported multiple times for the same subject with different CTCAE grades, the highest grade will be recorded.

2) Events will be summarized by frequency and percentage according to SOC and/or PT. Analysis will cover adverse events, serious adverse events, adverse reactions, severity of adverse events, and events leading to treatment discontinuation across dose groups.

3) Adverse events of special interest will be described and summarized in the Statistical Analysis Plan (SAP).

4) Incidence rates and reasons for death will be summarized and listed.

5) Changes in laboratory indicators, electrocardiograms, and physical examinations worsening post-trial compared to baseline will be analyzed and listed using frequencies and percentages.

XI. Study management

Clinical trial protocols and written informed consent forms, along with relevant materials directly related to participants, must be submitted to the ethics committee for written approval before the study can officially begin. Researchers must submit annual reports to the ethics committee regularly (based on the continuous review period specified in the approval). Researchers must notify the ethics committee in writing when a study is terminated and/or completed. Any changes in the study (such as amendments to the protocol and/or informed consent numbers) must be reported promptly to the ethics committee and cannot be implemented without their approval.

Researchers must provide approved informed consent forms to participants or their legal representatives and allow sufficient time for consideration before obtaining the signed informed consent. Updated versions of informed consent forms and written information must be provided to participants during their involvement in the study. Informed consent forms should be retained as essential documents of the clinical trial.

1. Approval of ethics review committees

Prior to implementing this protocol, it must be reviewed and approved by the hospital ethics committee. The research protocol, protocol amendments, informed consent form, and other relevant documents must be submitted to this ethics

committee. This clinical trial must adhere to the Helsinki Declaration, GCP issued by the CFDA, and relevant regulations. Approval from the hospital ethics committee is required before starting this study.

During the clinical study, any modifications to this trial protocol must be submitted to the ethics committee, and if necessary, other study documents should also be amended and submitted and/or approved as required by the ethics committee. The ethics committee must be notified when the trial ends.

2. Informed consent

Prior to initiation of any study procedures, prospective subjects will be provided with an informed consent form (ICF) that explains the risks and potential benefits of participation in the study. The language used in the ICF should be simple and understandable. The ICF should explicitly state that participation is voluntary and that subjects may withdraw from the study at any time without reprisal. Researchers may only enroll subjects after they have provided adequate information about the study, satisfactorily answered any questions, and allowed sufficient time for consideration, obtaining written consent from the subject or their legally authorized representative. All signed informed consent forms must be kept in the researcher's file or the subject's file.

Researchers are responsible for explaining the contents of the informed consent to subjects and obtaining written consent with a date from subjects or their legally authorized representatives before starting the study. After signing, researchers should provide subjects with a copy of the signed informed consent form. The process of informed consent must be documented in the trial's source documents.

3. Data collection

CRFs will be completed by the researcher, with each eligible case requiring completion of the CRF and entry into the Electronic Data Capture (EDC) system.

Completed case report forms will be reviewed by the principal investigator at each center and clinical monitor and kept by each center's researcher. Data entered into the EDC system will be submitted to relevant statistical units for data entry, management, and final statistical analysis.

4. Data management and quality control

This study will utilize an EDC system, where study data will be entered into electronic case report forms (eCRFs) by researchers or authorized personnel. Prior to initiation or data entry, researchers and authorized personnel will undergo appropriate training and appropriate security measures will be taken for the computer and other devices used.

Data entry into the eCRF should be completed as soon as possible during or after the visit to ensure that it reflects the latest status of the subjects participating in the study. To minimize differences in assessment of results by different evaluators, it is recommended that the same personnel complete the baseline and all subsequent efficacy and safety assessments for the same subject. Researchers must review the data to ensure the accuracy and correctness of all data entered into the eCRF. If certain assessments are not conducted during the study process or if certain information is unavailable, not applicable, or unknown, researchers should record this in the eCRF. Researchers should electronically sign verified data.

Monitors (CRAs) will review the eCRFs for completeness and consistency, comparing them with source documents to ensure consistency of key data. All data entry, corrections, and modifications will be the responsibility of the researcher or designated personnel. Changes to data in the eCRF will be recorded in the audit trail, including the reason for the change, the operator's name, modification time, and date. Roles and permissions for study center staff responsible for data entry will be predetermined. If there are data queries, the CRA or data manager will issue queries

in the EDC, and center staff will be responsible for responding. The EDC system will record an audit trail for queries, including the researcher's name, time, and date.

Unless otherwise specified, the eCRF will serve only as a data collection form and not as original source material. Original source documents are those used by researchers or hospitals, related to subjects, and which demonstrate the existence of subjects, inclusion/exclusion criteria, and participation in the study, including laboratory records, electrocardiogram results, pharmacy dispensing records, subject files, etc.

Researchers are responsible for maintaining all original documents and making them available for monitoring by CRAs during each visit. Additionally, regardless of the duration of a subject's participation in the study, researchers must submit a complete eCRF for each enrolled subject. Care must be taken to verify the protocol number and subject number for all supporting documents (such as laboratory records or hospital records) submitted with the eCRF and to remove all personal identifying information (including subject names) or make it unrecognizable to protect subject privacy. Researchers will electronically sign to attest their review of these records and the accuracy of the data in those records. Electronic signatures will be made using the researcher's user ID and password, with the system automatically attaching the date and time of the signature. Researchers are prohibited from sharing their user ID and password with others. If data in the eCRF needs to be changed, it should follow the workflow defined in the EDC system. All changes and reasons for changes will be recorded in the audit trail.

Adverse events and associated medical history/diagnoses will be coded. The coding dictionary will be described in the Clinical Study Report (CSR).

5. Study monitoring

Clinical monitoring of this study will be conducted by the sponsor or CRO authorized by the sponsor. Clinical monitors will conduct monitoring according to the

sponsor's or CRO's standard operating procedures and will have the same rights and responsibilities as sponsor monitors. Monitors will maintain regular communication with researchers and sponsors.

Before the start of the study, the monitor will evaluate the competence of each research center and report any issues related to facilities, technical equipment, or medical personnel to the sponsor. During the research process, the monitor will be responsible for monitoring whether the researcher has obtained written informed consent from all subjects and whether the data records are correct and complete. At the same time, the monitor will compare the data input into eCRF with the original data and inform the researchers of any errors or omissions that may have occurred. The inspector will also control the compliance of the research center's protocol, arrange the supply of research drugs, and ensure that the drugs are stored under appropriate conditions.

Monitoring visits will be conducted in accordance with relevant laws and regulations. Starting from the enrollment of subjects, each center will receive regular monitoring visits. After each visit to the researcher, the monitor should submit a written report to the sponsor.

6. **Protocol amendments**

Any necessary amendments to the protocol during the study will be communicated and agreed upon between the sponsor and investigators. The sponsor ensures timely submission of protocol amendments to regulatory authorities.

All protocol amendments are documented as protocol amendments. Any modifications to the protocol must be submitted to the ethics committee for approval or notification, as per committee requirements. If necessary, submissions should also be made to regulatory authorities for approval, and execution is subject to approval by the EC and regulatory authorities (if required), except for changes made to eliminate direct harm to trial subjects.

7. **Study termination**

The study may be terminated prematurely if it meets the following criteria:

1) Inaccurate or incomplete data records;

2) Poor compliance with the study protocol and regulatory requirements;

3) Adverse event rates or severity in this or other studies suggesting potential harm to subjects' health, prompting a change or discontinuation of drug development;

4) If the sponsor decides to discontinue supply of the investigational drug, adequate notice will be given to adjust subject treatments accordingly.

8. **Quality assurance**

During the study, the sponsor or its authorized representative may conduct quality assurance audits of study centers, study databases, and related research documents. Similarly, regulatory authorities may independently decide to inspect study centers, study databases, and related research documents. Investigators must promptly inform the sponsor upon receipt of notification of regulatory inspections.

The sponsor’s quality assurance department audits clinical trial institutions. Audits include: drug supply, required trial documents, records of informed consent processes, and consistency between case report forms and original documents, among others. Audit scope and content may be expanded as necessary. Following reasonable notice, investigators should allow authorized audit personnel appointed by the sponsor to conduct trial-related audits and inspections by regulatory authorities. The primary purpose of audits or inspections is to ensure that trial subjects' rights and health are protected, that the informed consent process is properly conducted, and that handling and reporting of all data related to investigational drug evaluations are consistent with pre-planned arrangements, protocols, facilities, ethical standards, GCP, and applicable

regulatory requirements. Investigators should have direct access to all trial documents, original records, and raw data.

9. Confidentiality

Investigators agree to grant the sponsor, CRO, and relevant authorized regulatory authorities direct access to all study-related documents, including subject medical records.

ICFs will include (or in some cases, along with the use of independent documents) relevant information on data protection and privacy protection. Precautions are taken to ensure the confidentiality of documents to prevent identification of subjects. However, in special circumstances, certain personnel may have access to a subject's data and personal identification codes. For example, in cases of medical emergencies, the sponsor, its designated physician, or investigators may be aware of subject identification codes and have access to the subject's genetic data. Additionally, relevant regulatory authorities require access to relevant documents.

10. Retention of study records

Documents in clinical trials (protocols and protocol amendments, completed eCRFs, signed ICFs, etc.) must be retained and managed in accordance with GCP requirements. Study centers should retain these documents for 5 years after the study ends. Study documents should be reasonably stored for future access or data traceability, considering security and environmental risks.

No study documents may be destroyed without written permission from the sponsor and investigators. Study documents may only be transferred to other parties who comply with document retention requirements or to other locations that meet these requirements, after notifying the sponsor and obtaining their written consent.

XII. Protocol compliance

Investigators undertake to avoid protocol deviations to the best of their ability. If investigators believe that a deviation from the protocol could improve the conduct of the trial, protocol amendments must be considered. However, such amendments can only be implemented after approval by the Medical Ethics Committee. All significant protocol deviations will be documented and reported in the clinical trial report.

XIII. Ethical principles and requirements for clinical research

Clinical research will adhere to relevant regulations such as the World Medical Association's Declaration of Helsinki and the National Health and Family Planning Commission of the People's Republic of China's "Ethical Review Measures for Biomedical Research Involving Human Subjects". Specific implementation includes informed consent, privacy protection, free and compensated research, risk control, protection of special subjects, and principles and requirements for compensation related to research-related harm. Before the study begins, the trial protocol must be approved by the Ethics Review Committee to proceed with clinical research. Before enrollment in this study, investigators are responsible for fully and comprehensively informing subjects and/or their legal representatives about the purpose, procedures, and potential risks of the study, and obtaining their written informed consent. Subjects should be informed that their participation in the clinical study is entirely voluntary, and they have the right to refuse to participate or to withdraw from the study at any stage without discrimination or retaliation, with no impact on their medical treatment and rights. The informed consent form should be retained as part of the clinical research documents, ensuring the protection of subjects' personal privacy and data confidentiality.
