# Supplementary material for: Efficacy, safety and single-cell analysis of neoadjuvant immunochemotherapy in locally advanced oral squamous cell carcinoma: a phase II trial
Source: Nat Commun. 2025 Apr 28;16:3968. doi: 10.1038/s41467-025-59004-w (PMC12037888; doi:10.1038/s41467-025-59004-w)
Supplement: Supplementary file 2 — Reporting Summary [file 41467_2025_59004_MOESM2_ESM.pdf]

nature portfolio

Corresponding author(s): Lei Liu

Last updated by author(s): Mar 9, 2025

Reporting Summary

Nature Portfolio wishes to improve the reproducibility of the work that we publish. This form provides structure for consistency and transparency in reporting. For further information on Nature Portfolio policies, see our Editorial Policies and the Editorial Policy Checklist.

Statistics

For all statistical analyses, confirm that the following items are present in the figure legend, table legend, main text, or Methods section.

n/a	Confirmed	
☐	☒	The exact sample size (*n*) for each experimental group/condition, given as a discrete number and unit of measurement
☐	☒	A statement on whether measurements were taken from distinct samples or whether the same sample was measured repeatedly
☐	☒	The statistical test(s) used AND whether they are one- or two-sided<br>*Only common tests should be described solely by name; describe more complex techniques in the Methods section.*
☐	☒	A description of all covariates tested
☐	☒	A description of any assumptions or corrections, such as tests of normality and adjustment for multiple comparisons
☐	☒	A full description of the statistical parameters including central tendency (e.g. means) or other basic estimates (e.g. regression coefficient) AND variation (e.g. standard deviation) or associated estimates of uncertainty (e.g. confidence intervals)
☐	☒	For null hypothesis testing, the test statistic (e.g. *F*, *t*, *r*) with confidence intervals, effect sizes, degrees of freedom and *P* value noted<br>*Give P values as exact values whenever suitable.*
☒	☐	For Bayesian analysis, information on the choice of priors and Markov chain Monte Carlo settings
☒	☐	For hierarchical and complex designs, identification of the appropriate level for tests and full reporting of outcomes
☐	☒	Estimates of effect sizes (e.g. Cohen's *d*, Pearson's *r*), indicating how they were calculated

Our web collection on statistics for biologists contains articles on many of the points above.

Software and code

Policy information about availability of computer code

Data collection	1. NovoCyte Advanteon Flow Cytometer (Agilent Technologies, Inc.) was used for collecting flow cytometry data.<br>2. Vectra Polaris Software (Akoya Biosciences) were used for collecting multiplex fluorescence immunohistochemistry data.<br>3. Qseq400 (BiOptic Inc.) and NextSeq Software (Illumina) were used for collecting whole exome sequencing data.
Data analysis	1. NovoCyte Advanteon Flow Cytometer (Agilent Technologies, Inc.) was used for flow cytometry data analysis.<br>2. QuPath (QuPath-0.4.3) was used for multiplex fluorescence immunohistochemistry data data analysis.<br>3. Cell Ranger software (version 6.0.1) was used for FASTQ files data analysis. Seurat (version 4.0.0) R package and DoubletFinder package (version 2.0.3) were used for UMI count matrix analysis. R package VISION (version 3.0.1) was used for GO enrichment pathway enrichment analysis. Monocle package (version 2.18.0) was used for single-cell trajectory analysis. CellChat (v 1.1.0) was used for cell-cell communication analysis. Source code for data analysis is available on GitHub (https://github.com/sdzxzh/SC-neoadjuvant).

For manuscripts utilizing custom algorithms or software that are central to the research but not yet described in published literature, software must be made available to editors and reviewers. We strongly encourage code deposition in a community repository (e.g. GitHub). See the Nature Portfolio guidelines for submitting code & software for further information.

Data

Policy information about availability of data

All manuscripts must include a data availability statement. This statement should provide the following information, where applicable:

- Accession codes, unique identifiers, or web links for publicly available datasets
- A description of any restrictions on data availability
- For clinical datasets or third party data, please ensure that the statement adheres to our policy

The trial protocol is available as Supplementary Note in the Supplementary Information file. The anonymized patient data supporting this study's findings, including clinical demographics, tumor characteristics, safety assessments, radiographic/pathological evaluations from neoadjuvant therapy are provided within the article and supplementary materials. The raw sequence data targeted WES and scRNA-seq/scTCR-seq have been deposited in Genome Sequence Archive in BIG Data Center, Beijing Institute of Genomics (BIG, http://bigd.big.ac.cn/gsa-human/) with Project Accession No. PRJCA034157 and GSA Accession No. HRA009876. The raw sequencing data contain information unique to individuals are available under controlled access, authorization by the Clinical Research Ethics Board is required as per institutional policy prior to any disclosure of participants' genomic data and individual-level clinical data, which will be made available for scholarly research objectives upon submission of a justified application to the corresponding author via liuleihx@gmail.com. The TCGA database is available at https://portal.gdc.cancer.gov/. The raw flow cytometry data and multiplex immunofluorescence staining images data generated in this study have been deposited in the Zenodo under accession DOI: 10.5281/zenodo.14993113. The other data are available within the manuscript, Supplementary Information, and Source file. Source data are provided with this paper.

Research involving human participants, their data, or biological material

Policy information about studies with human participants or human data. See also policy information about sex, gender (identity/presentation), and sexual orientation and race, ethnicity and racism.

Reporting on sex and gender	The study protocol did not include pre-specified sex- or gender-based analyses. Patients were included regardless of sex or gender.
Reporting on race, ethnicity, or other socially relevant groupings	All patients enrolled in this study were Chinese people, whithout any race, ethnicity or other sociallyrelevant groupings.
Population characteristics	Inclusion criteria included: aged 18-75 years; newly diagnosed, histologically confirmed, resectable stage III-IVB OSCC according to the 8th Union for International Cancer Control and American Joint Committee on Cancer (stage IVB patients were eligible only if they had no skull base invasion, no internal carotid artery encasement, and were resectable after strict evaluation by surgeons before enrollment); Eastern Cooperative Oncology Group performance status of 0 to 1; and adequate organ function. Exclusion criteria included: distant metastasis; refusal of surgery; intent for palliative treatment; previous treatments with chemotherapy, radiotherapy, targeted therapy, anti-PD-L1/anti-PD1 agents or surgery for primary or metastatic nodes (except biopsy); previous malignancy; pregnancy or lactation; severe comorbidities; autoimmune diseases; and intolerance to immunotherapy.
Recruitment	Patients that presented with initial diagnosis of resectable stage III-IVB locally advanced oral squamous cell carcinoma, either at our center or referred from other centers, and who were potentially eligible for this study were were informed of this and other studies for which they were eligible. patients who were deemed eligible were informed about the aims of the study, the possible adverse events, and the procedures and possible hazards to which he/she would be exposed. Patients were followed by the multidisciplinary team (MDT) in our center.
Ethics oversight	This single-arm, phase II trial was conducted at West China Hospital, Sichuan University and was approval by the ethics committee of our center. The trial was registered with the Chinese Clinical Trial Registry (ChiCTR2200066119) on November 24, 2022, and adhered to the Declaration of Helsinki and Good Clinical Practice guidelines. All patients signed informed consent before enrollment.

Note that full information on the approval of the study protocol must also be provided in the manuscript.

Field-specific reporting

Please select the one below that is the best fit for your research. If you are not sure, read the appropriate sections before making your selection.

☒ Life sciences ☐ Behavioural & social sciences ☐ Ecological, evolutionary & environmental sciences

For a reference copy of the document with all sections, see nature.com/documents/nr-reporting-summary-flat.pdf

Life sciences study design

All studies must disclose on these points even when the disclosure is negative.

Sample size	We used a Simon two-stage design with an one-sided α value of 0.05 and a power of 80% to assess the efficacy of the new NAIC regimen of camrelizumab plus nab-paclitaxel and cisplatin. We assumed that the new NAIC regimen could increase the MPR to 55% compared to a historical control of 27.7% with docetaxel 75 mg/m2, cisplatin 75 mg/m2, and fluorouracil 750 mg/m2. In the first stage, 9 patients were required. If more than 3 patients achieved MPR, recruitment would proceed to the second stage with an additional 19 patients, the total sample size is up to 28 patients in this two-stage Phase 2 study. Considering a dropout rate of 10%, a total of 31 patients were needed in the

	study. If more than 11 patients achieved MPR among 28 patients eligible for the MPR evaluation, indicating the new regimen was worth for further exploration.
Data exclusions	Primary and secondary endpoints were analyzed in the per protocol population. All patients who did not receive at least one dose of study drugs (camrelizumab, nab-paclitaxel, and cisplatin) were excluded from the per protocol population (n=1). For the exploratory analyses aiming to dissect the tumor microenvironment, patients who did not receive any of the intended treatment cycles of combination camrelizumab plus chemotherapy were excluded (n=1).
Replication	For cellular experiments, each single measurement was performed in five repetitions and the results were consistently reproducible. Replication is not applicable for clinical data. Translational experiments on human samples were not replicated due to limited material.
Randomization	Patients were not randomized, all patients received 2 cycles of NAIC, with camrelizumab 200 mg and nab-paclitaxel 260 mg/m2 administered intravenously on days 1 and 22 and cisplatin 75 mg/m2 (divided into 3 days) on days 1-3 and days 22-24. Surgery was performed within 2-4 weeks after NAIC. Adjuvant radiotherapy or chemoradiotherapy was initiated within 6 weeks after surgery. Maintenance immunotherapy with camrelizumab 200 mg for 6 cycles was planned after surgery
Blinding	No blinding was performed as all patients received 2 cycles of NAIC, with camrelizumab, nab-paclitaxel and cisplatin. Followed by surgery, adjuvant radiotherapy or chemoradiotherapy, and maintenance camrelizumab therap

Reporting for specific materials, systems and methods

We require information from authors about some types of materials, experimental systems and methods used in many studies. Here, indicate whether each material, system or method listed is relevant to your study. If you are not sure if a list item applies to your research, read the appropriate section before selecting a response.

Materials & experimental systems

n/a	Involved in the study
☐	☒ Antibodies
☐	☒ Eukaryotic cell lines
☒	☐ Palaeontology and archaeology
☐	☒ Animals and other organisms
☐	☒ Clinical data
☒	☐ Dual use research of concern
☒	☐ Plants

Methods

n/a	Involved in the study
☒	☐ ChIP-seq
☐	☒ Flow cytometry
☒	☐ MRI-based neuroimaging

Antibodies

Antibodies used	Antibodies used in multiplex immunfluorescence staining for tumor in patients:<br>Panel 1: CD3 (CST, dilution 1:200, catalog no.85061), CD8 (CST, dilution 1:200, catalog no.853365), CD4 (CST, dilution 1:100, catalog no.48274), CD20 (Maixin, dilution 1:200, catalog no. kit-0001), FoxP3 (CST, dilution 1:100, catalog no.98377), Pan-CK (Abcam, dilution 1:200, catalog no.7753).<br>Panel 2: CD11c (CST, dilution 1:200, catalog no.455815), CD68 (CST, dilution 1:200, catalog no.76437), CD163 (Maixin, dilution 1:100, catalog no. MAB-0869), CD56 (Maixin, dilution 1:100, catalog no. MAB-0743), Pan-CK (Abcam, dilution 1:200, catalog no.7753), Gr-1 (Novus, dilution 1:100, catalog no. NBP2-00441).<br>Panel 3: CXCR5 (Abclonal, dilution 1:200, catalog no.A8950), CXCL13 (HUABIO, dilution 1:200, catalog no.HA722117), CD 20 (Maixin, dilution 1:200, catalog no. kit-0001), CD21 (Maixin, dilution 1:200, catalog no. RMA-0811), CD8 (CST, dilution 1:200, catalog no.853365), CD4 (CST, dilution 1:100, catalog no.48274).<br>DAPI (Sigma, dilution 1:1000, catalog no.D9542)<br><br>Antibodies used in multiplex immunfluorescence staining for tumor in mice:<br>anti-CD4 (1:200, CST, 25229), anti-CD8 (1:1000, Abcam, 217344), and anti-CD20 (1:100, Invitrogen, PA5-16701).<br><br>T cells and B cells detection flow cytometry antibodies for tumor in patients:<br>anti-CD45 (1:100, Absin, abs182374-100T), anti-CD3 (1:100, BD Pharmingen, 563024), anti-CD4 (1:100, BD Pharmingen, 566407), anti-CD8 (1:100, BD Pharmingen, 563234), anti-CXCL13 (1:100, Thermo, 17-7981-82), and anti-CD19 (1:100, BD Pharmingen, 562701).
Validation	The validation of all of the antibodies depends on product data sheet and published literature.<br><br>Antibodies used in multiplex immunfluorescence staining for tumor in patients:<br>1. CD3 (CST, dilution 1:200, catalog no.85061), https://www.cellsignal.cn/products/primary-antibodies/cd3e-d7a6e-xp-rabbit-mab/85061<br>2. CD8 (CST, dilution 1:200, catalog no.85336), https://www.cellsignal.cn/products/primary-antibodies/cd8a-d8a8y-rabbit-mab/85336<br>3. CD4 (CST, dilution 1:100, catalog no.48274), https://www.cellsignal.cn/products/primary-antibodies/cd4-ep204-rabbit-mab/48274<br>4. CD20 (Maixin, dilution 1:200, catalog no. kit-0001), http://www.maxim.com.cn/sitecn/dklkthdklkt/7028.html

FoxP3 (CST, dilution 1:100, catalog no.98377), https://www.cellsignal.cn/products/primary-antibodies/foxp3-d2w8e-rabbit-mab/98377
5. Pan-CK (Abcam, dilution 1:200, catalog no.7753). https://www.abcam.cn/products/primary-antibodies/pan-cytokeratin-antibody-c-11-ab7753.html
6. CD11c (CST, dilution 1:200, catalog no.45581), https://www.cellsignal.cn/products/primary-antibodies/cd11c-d3v1e-xp-rabbit-mab/45581
7. CD68 (CST, dilution 1:200, catalog no.76437), https://www.cellsignal.cn/products/primary-antibodies/cd68-d4b9c-xp-rabbit-mab/76437
8. CD163 (Maixin, dilution 1:100, catalog no. MAB-0869), http://www.maxim.com.cn/sitecn/dklkthdklkt/7534.html
9. CD56 (Maixin, dilution 1:100, catalog no. MAB-0743), http://www.maxim.com.cn/sitecn/dklkthdklkt/7434.html
10. CXCR5 (Abclonal, dilution 1:200, catalog no.A8950), https://abclonal.com.cn/catalog/A8950
11. CXCL13 (HUABIO, dilution 1:200, catalog no.HA722117), https://huabio.cn/products/CXCL13-antibody-HA722117
12. CD21 (Maixin, dilution 1:200, catalog no. RMA-0811), http://www.maxim.com.cn/sitecn/dklkthdklkt/7428.html
13. Gr-1 (Novus, dilution 1:100, catalog no. NBP2-00441), https://www.novusbio.com/products/ly-6g-ly-6c-antibody-rb6-8c5_nbp2-00441
14. DAPI (Sigma, dilution 1:1000, catalog no.D9542), https://www.sigmaaldrich.cn/CN/zh/product/sigma/d9542

Antibodies used in multiplex immunfluorescence staining for tumor in mice:
1. anti-CD4 (CST, 1:200, 25229), https://www.cellsignal.cn/products/primary-antibodies/cd4-d7d2z-rabbit-mab/25229
2. anti-CD8 (Abcam, 1:1000, 217344), https://www.abcam.cn/products?keywords=217344
3. anti-CD20 (Invitrogen, 1:100, PA5-16701). https://www.fishersci.com/shop/products/cd20-polyclonal-antibody-invitrogen-1/PIPA516701?searchHijack=true&searchTerm=PA5-16701&searchType=RAPID&matchedCatNo=PA5-16701

T cells and B cells detection flow cytometry antibodies for tumor in patients:
1. anti-CD45 (1:100, Absin, abs182374-100T), https://www.absin.cn/fitc-rat-anti-mouse-cd45/abs182374.html
2. anti-CD3 (1:100, BD Pharmingen, 563024), https://www.bdbiosciences.com/zh-cn/search-results?searchKey=563024
3. anti-CD4 (1:100, BD Pharmingen, 566407), https://www.bdbiosciences.com/zh-cn/search-results?searchKey=566407
4. anti-CD8 (1:100, BD Pharmingen, 563234), https://www.bdbiosciences.com/zh-cn/search-results?searchKey=563234
5. anti-CXCL13 (1:100, Thermo, 17-7981-82), https://www.thermofisher.cn/cn/zh/antibody/product/CXCL13-Antibody-clone-DS8CX13-Monoclonal/17-7981-82
6.anti-CD19 (1:100, BD Pharmingen, 562701), https://www.bdbiosciences.com/zh-cn/search-results?searchKey=562701

Eukaryotic cell lines

Policy information about cell lines and Sex and Gender in Research

Cell line source(s)	The mouse squamous cell carcinoma cell line (SCC7) was kindly provided by Department of Head and Neck Oncology, West China Hospital of Stomatology, State Key Laboratory of Oral Diseases, National Clinical Research Centre for Oral Diseases, Sichuan University.
Authentication	No
Mycoplasma contamination	No
Commonly misidentified lines (See ICLAC register)	No

Animals and other research organisms

Policy information about studies involving animals; ARRIVE guidelines recommended for reporting animal research, and Sex and Gender in Research

Laboratory animals	C3H mice (male, 6-8 weeks) were purchased from the BEIJING HFK BIOSCIENCE CO.,LTD. All mice were acclimated for 7 days before experiment. Mice were kept in pathogen-free cages with well ventilated shelves with a controlled temperature (22 - 24 ℃) and light cycle (12 h light, 12 h dark), and were supplied with sufficient food and water. Animal experiments were approved by the Institutional Animal Care and Treatment Committee of Sichuan University (Chengdu, China). Animals were housed in the SPF laboratory animal room of the State Key Laboratory of Biotherapy of Sichuan University (Chengdu, China). In the study, all mice were euthanized by cervical dislocation.
Wild animals	No wild animals were used in this study.
Reporting on sex	In this study, all mice used were male, mainly based on the published literatures.
Field-collected samples	No field-collected samples were used.
Ethics oversight	Animal experiments were approved by the Institutional Animal Care and Treatment Committee of Sichuan University (Chengdu, China).

Note that full information on the approval of the study protocol must also be provided in the manuscript.

Clinical data

Policy information about clinical studies

All manuscripts should comply with the ICMJEguidelines for publication of clinical research and a completedCONSORT checklist must be included with all submissions.

Clinical trial registration	The trial was registered with the Chinese Clinical Trial Registry (ChiCTR2200066119) on November 24, 2022.
Study protocol	The study protocol is available as Supplementary Note in the Supplementary Information file.
Data collection	Between February 1, 2023 and May 31, 2024, 35 patients were screened and 33 patients were enrolled at West China Hospital, Sichuan University. And the follow-up time is between February 1, 2023 and November 30, 2024.
Outcomes	The primary endpoints were MPR (defined as %RVT ≤10%) rate and safety. The secondary endpoints included pathological complete response (pCR, defined as no tumor cells on H&E slides) rate, objective response rate (ORR), disease-free survival (defined as the time from surgery to the first documented disease recurrence or death from any cause, with data censored for patients who remained alive or free of disease recurrence at the last follow-up), and overall survival (defined as the time from initial treatment to death from any cause, with data censored for patients who remained alive at the last follow-up). AEs were graded by the Common Terminology Criteria for Adverse Events version 5.0. Surgery-related complications were graded by the Clavien-Dindo system.

Plants

Seed stocks	This study did not involve plants.
Novel plant genotypes	This study did not involve plants.
Authentication	This study did not involve plants.

Flow Cytometry

Plots

Confirm that:

- [x] The axis labels state the marker and fluorochrome used (e.g. CD4-FITC).
- [x] The axis scales are clearly visible. Include numbers along axes only for bottom left plot of group (a 'group' is an analysis of identical markers).
- [x] All plots are contour plots with outliers or pseudocolor plots.
- [x] A numerical value for number of cells or percentage (with statistics) is provided.

Methodology

Sample preparation	The harvested mice tumors were cut up and digested into single cell suspension. After red blood cell lysis, cells were stained with anti-CD45 (1:100, Absin, abs182374-100T), anti-CD3 (1:100, BD Pharmingen, 563024), anti-CD4 (1:100, BD Pharmingen, 566407), anti-CD8 (1:100, BD Pharmingen, 563234), anti-CXCL13 (1:100, Thermo, 17-7981-82), and anti-CD19 (1:100, BD Pharmingen, 562701).
Instrument	FovoCyte Advanteon Flow Cytometer (Agilent Technologies, Inc.) was used to analyze the stained cells.
Software	Novo Express software (ACEA Biosciences) software v10.9.0 were used for data analyses.
Cell population abundance	CD45-positive cells accounts for about 90% of the lymphocytes. CD3-positive cells accounts for about 30% in CD45-positive cells.<br>CD4 accounts for about 50% of the CD3-positive cells. CD8 accounts for about 20% of the CD3-positive cells.<br>CD19 accounts for about 50% of the CD3-negative cells. CXCL13-positive cells accounts for 10% in CD4-positve cells.
Gating strategy	Flow cytometry was widely used in this study.<br>CXCL13+ T cells were defined as CD45+CD3+ CD4+CXCL13+;<br>CD19+ B cells were defined as CD45+CD3- CD19+;<br>CD8+ T cells were defined as CD45+CD3+CD8+.

- [x] Tick this box to confirm that a figure exemplifying the gating strategy is provided in the Supplementary Information.
